# Generic decoding of seen and imagined objects using hierarchical visual features

**DOI:** 10.1038/ncomms15037

**Published:** 2017-05-22

**Authors:** Tomoyasu Horikawa, Yukiyasu Kamitani

**Affiliations:** 1 ATR Computational Neuroscience Laboratories, 2-2-2 Hikaridai, Seika, Soraku, Kyoto 619-0288, Japan; 2 Graduate School of Informatics, Kyoto University, Yoshida-honmachi, Sakyo-ku, Kyoto 606-8501, Japan

## Abstract

Object recognition is a key function in both human and machine vision. While brain decoding of seen and imagined objects has been achieved, the prediction is limited to training examples. We present a decoding approach for arbitrary objects using the machine vision principle that an object category is represented by a set of features rendered invariant through hierarchical processing. We show that visual features, including those derived from a deep convolutional neural network, can be predicted from fMRI patterns, and that greater accuracy is achieved for low-/high-level features with lower-/higher-level visual areas, respectively. Predicted features are used to identify seen/imagined object categories (extending beyond decoder training) from a set of computed features for numerous object images. Furthermore, decoding of imagined objects reveals progressive recruitment of higher-to-lower visual representations. Our results demonstrate a homology between human and machine vision and its utility for brain-based information retrieval.

## Abstract (web summary)

Machine learning algorithms can decode objects that people see or imagine from their brain activity. Here the authors present a predictive decoder combined with deep neural network representations that generalizes beyond the training set and correctly identifies novel objects that it has never been trained on.

Brain decoding through machine learning analysis of functional magnetic resonance imaging (fMRI) activity has enabled the interpretation of mental contents, including what people see^1^,^2^, remember^3^,^4^,^5^,^6^,^7^, imagine^8^,^9^,^10^,^11^,^12^ and dream^13^. Most previous studies have relied on a classification-based approach, where a statistical classifier (decoder) is trained to learn a relationship between fMRI patterns and the target contents to be decoded. Such approaches entail a fundamental constraint on the number of possible outputs. Namely, the outputs are limited to the classes used for decoder training, preventing the decoder from predicting any classes that are not used in training.

Recent studies have overcome this limitation by designing decoders for retinotopically organized, image-level features^14^,^15^,^16^. This method enables the decoding of novel visual images not presented during training sessions. Kay *et al*.^14^ built an encoding model consisting of retinotopically organized Gabor wavelet filters. They used a visual image database and the predicted brain activities produced by an encoding model. The measured brain activity was then decoded by determining the visual image in the database corresponding to the predicted brain activity most similar to the measured brain activity. This technique has also been used to identify remembered artworks from the early visual cortical activity^17^. Miyawaki *et al*.^16^ constructed a modular decoding model consisting of multi-scale local decoders (modules) that predicted the contrast of local image patches. The model enabled reconstruction of arbitrary visual images from brain activity by combining the outputs of the local decoders despite having been trained with brain activity for a small number of random images.

While visual image identification^14^,^15^,^17^ and reconstruction^16^ are suitable for decoding according to image-based similarity, they do not provide explicit information regarding the object a person is seeing or imagining. The possible objects we may see or imagine in daily life are countless, and object-based information is often more directly relevant to our visually guided behaviour than image-based information. Establishing methods for decoding generic object categories from brain activity would provide practical benefits for technologies utilizing information decoded from brain activity and may increase understanding of the way the human brain represents a vast number of objects.

In this study, we aim to decode seen and imagined object categories, including those not used in decoder training from fMRI signals measured while subjects either viewed or imagined object images. We extended the modular decoding approach originally developed for visual image reconstruction^16^ to the decoding of generic object categories.

To tailor the modular decoding approach to the objectives of this study, we assumed that an object category can be represented by a set of visual features with several invariances. These features correspond to those proposed for the object recognition challenge in machine vision^18^,^19^,^20^,^21^,^22^,^23^,^24^ (Fig. 1a), which aims at enabling a computer to recognize objects in images according to their category names. The selection of visual features is a critical aspect of this approach because even if images depict the same object, they do not necessarily have pixel-wise similarity as a result of varying rotation, scale, position and other visual attributes. Thus objects may be more suitably represented using mid- or high-level visual features which are invariant to such image differences rather than the low-level features (for example, local contrast^13^ or Gabor wavelet filter^14^,^15^,^17^) used for visual image reconstruction and identification.

We tested a total of 13 candidates of visual feature types/layers constructed from four models (Fig. 1a, see Methods section): a convolutional neural network (CNN) model^20^ (CNN1–CNN8), HMAX model^21^,^22^,^23^ (HMAX1–HMAX3), GIST^24^, and scale invariant feature transform^18^ combined with the ‘Bag of Features'^19^ (SIFT+BoF). Some of the models emulate the hierarchical structure of the human visual system (CNN and HMAX), while others are designed for scene recognition (GIST) and object recognition (SIFT+BoF) in machine vision. These visual feature types/layers have multiple levels of complexity, and it has been reported that representations of these model outputs are statistically similar to visual cortical activity^21^,^22^,^25^,^26^,^27^,^28^,^29^,^30^.

Using these visual features, we present a new approach called ‘generic object decoding,' in which arbitrary object categories are decoded from human brain activity (Fig. 1b). We used the online image database, ImageNet^31^, and trained regression models (decoders) to predict visual features extracted by the computational models from brain activity recorded by fMRI while subjects viewed natural images (150 categories). The trained decoders were then used to predict feature vectors of seen and imagined objects that were not used in decoder training from the fMRI activity patterns. By comparing the predicted feature vector with the category-average feature vectors calculated from images in the image database, we identify seen and imagined object categories from those defined in a database (15,372 categories in ImageNet^31^). Because arbitrary object categories are represented in this feature space, the identified categories are not limited to those used in training.

Here we first demonstrate that visual feature values of seen objects calculated by the computational models can be predicted from multiple brain areas, showing tight associations between hierarchical visual cortical areas and the complexity levels of visual features. In addition, we show that stimulus-trained decoders can be used to decode visual features of imagined objects, providing evidence for the progressive recruitment of hierarchical neural representations in a top-to-bottom manner. Finally, we test whether the features predicted from brain activity patterns are useful for identifying seen and imagined objects for arbitrary categories.

## Results

### Generic object decoding

**Figure 1:**
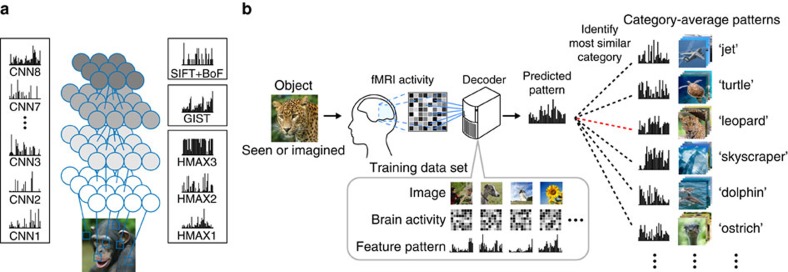
Generic object decoding. (**a**) Visual feature extraction from natural images using computational models. Visual features were calculated from natural images using CNN (CNN1–8), HMAX (HMAX1–3), GIST and SIFT+BoF. (**b**) Overview of generic object decoding. fMRI activity was measured while subjects viewed natural images. Decoders were trained to predict the values of the visual features for presented images/objects from multi-voxel fMRI signals. Given measured fMRI activity, a feature vector was predicted and it is used to identify the seen or imagined object by comparing it with the feature vectors of numerous objects in an annotated image database including those not used for decoder training.

Our objective was to decode arbitrary object categories (not included in model training) from human brain activity measured using fMRI. First, we extracted feature values from object images using a total of 13 visual feature types/layers (CNN1–8, HMAX1–3, GIST and SIFT+BoF; ∼1,000 units for each feature type/layer). We thereby represented an object image by a feature vector of each feature type/layer (Fig. 1a). Second, decoders were trained to predict the vectors of visual features of seen objects from brain activity patterns (Fig. 1b). Third, using the trained decoders, a feature vector was predicted from brain activity measured while seeing or imagining an object that was not used in decoder training. Finally, the predicted feature vector was used to identify a seen/imagined object by calculating the similarity between the predicted and the category-average feature vectors calculated from an annotated image database.

**Figure 2:**
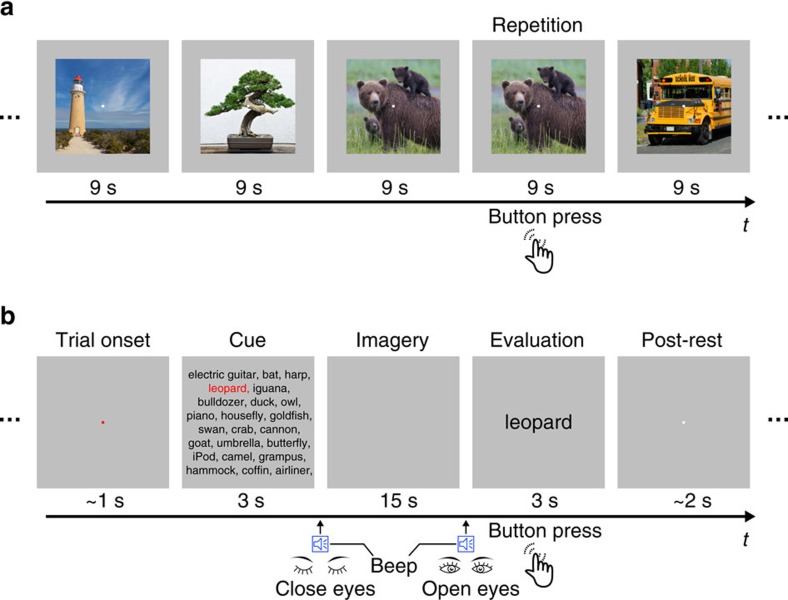
Experimental design. (**a**) Image presentation experiment. Images were presented in the centre of the display with a central fixation spot. The colour of the fixation spot changed from white to red for 0.5 s before each stimulus block started to indicate the onset of the block. Subjects maintained steady fixation throughout each run and performed a one-back repetition detection task on the images, responding with a button press for each repetition. (**b**) Imagery experiment. The onset of each trial was marked by a change in the fixation colour. Cue stimuli composed of an array of object names were visually presented for 3 s. The onset and the end of the imagery periods were signalled by auditory beeps. After the first beep, the subjects were instructed to imagine as many object images as possible pertaining to the category indicated by red letters. They continued imagining with their eyes closed (15 s) until the second beep. Subjects were then instructed to evaluate the vividness of their mental imagery (3 s). Note that the actual cue consisted of an array of 50 object names, while only subsets of the words are depicted in this figure because of space limitations.

To test the feasibility of generic decoding of seen and imagined objects from brain activity, we conducted two fMRI experiments: an image presentation experiment, and an imagery experiment (Fig. 2). In the image presentation experiment, fMRI signals were measured while subjects viewed a sequence of object images (Fig. 2a). The image presentation experiment consisted of two sessions: the training image session and the test image session. In the training image session, 1,200 images from 150 object categories (8 images from each category) were each presented once. In the test image session, 50 images from 50 object categories (one image from each category) were each presented 35 times. In the imagery experiment, fMRI signals were measured while subjects imagined about 1 of the 50 object categories (10 times for each category), which were the same as those in the test image session (Fig. 2b). The categories in the test image session and the imagery experiment were not used in the training image session. While we show results with fMRI signals averaged across all trials (35 trials for the test image session and 10 trials for the imagery experiment), quantitatively similar results were obtained with a much smaller number of averaged samples (see Supplementary Information).

We performed our analysis for each combination of feature types/layers and brain regions of interest (ROIs; V1–V4, the lateral occipital complex (LOC), fusiform face area (FFA), parahippocampal place area (PPA), lower visual cortex (LVC; V1–V3), higher visual cortex (HVC; covering regions around LOC, FFA and PPA) and the entire visual cortex (VC; covering all of the visual subareas listed above); see Methods section and Supplementary Fig. 1 for definitions of the ROIs).

A set of linear regression functions (sparse linear regression (SLR) model^32^) was used to predict visual feature vectors (∼1,000 feature units for each feature type/layer; see Methods section) from the fMRI signals in each ROI. A unit decoder was trained to predict the values of the feature vectors calculated from the viewed images, using fMRI signals from the training image session (that is, ∼1,000 decoders for each feature type/layer). The trained decoders were then used to predict the vectors of each feature type/layer for the test object categories from measured fMRI signals in the test image session and the imagery experiment.

### Image feature decoding

**Figure 3:**
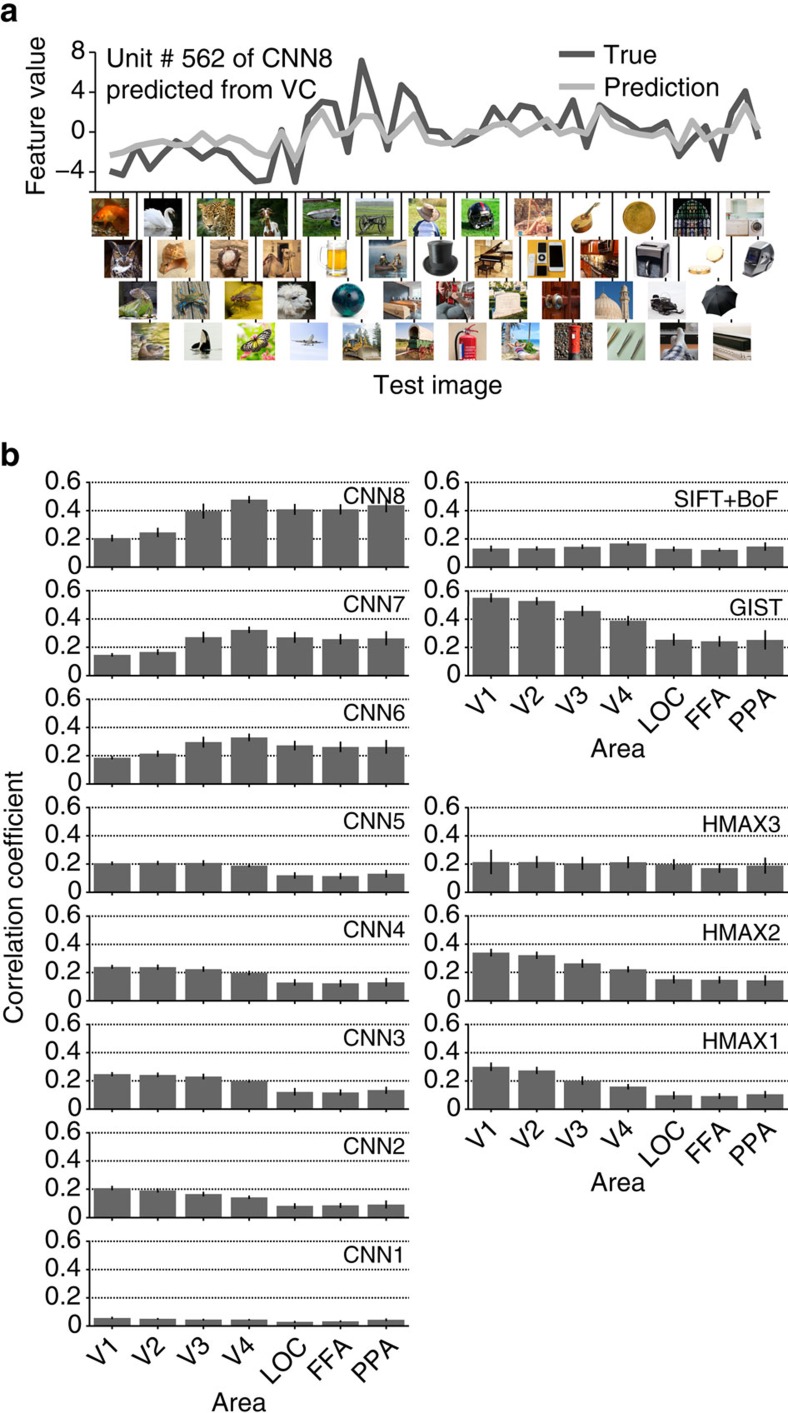
Image feature decoding and the homology of CNN and the human brain. (**a**) Example of a feature unit. True (black) and predicted (light grey) feature values for 50 test images are shown for Unit #562 of CNN Layer 8 (CNN8) predicted from the whole VC. (**b**) Image feature decoding accuracy. Mean decoding accuracies are shown for each combination of the feature type/layer and ROI (error bars, 95% confidence interval (CI) across five subjects).

We first investigated whether we could decode the values of visual feature vectors for presented images from brain activity. Decoding accuracy was evaluated by the correlation coefficient between true and predicted feature values of each feature unit (Fig. 3a). Correlation coefficients were averaged across the units in each feature type/layer for multiple ROIs and then averaged across five subjects. Because the distribution of feature values and the number of feature units of the original population differed between feature types/layers, interpreting decoding accuracy differences across feature types/layers was difficult. For example, when accuracy was evaluated by normalized root mean square errors, the overall pattern of accuracy across feature types/layers differed from the pattern derived from correlations (Supplementary Fig. 2). Therefore, in the following we focussed on the pattern of accuracies across ROIs in each feature type/layer.

Figure 3b shows the decoding accuracy for features of presented images in multiple ROIs. The predicted feature values positively correlated with the true values for all feature–ROI combinations (one-sided *t*-test after Fisher's *z*-transform, uncorrected *P*<0.05). Interestingly, the choice of feature types/layers and ROIs affected the accuracy pattern. As observed in the results for the CNN layers, higher-order features tended to be better predicted from fMRI signals in higher rather than lower ROIs, and lower-order features tended to be better predicted from fMRI signals in lower rather than higher ROIs (analysis of variance (ANOVA), interaction between layer and ROI, *P*<0.01). Similar tendencies were also observed with HMAX (ANOVA, interaction between feature type and ROI, *P*<0.01). Such differences were also observed in the decoding accuracies obtained from LVC and HVC (Supplementary Fig. 3). These results reveal a tight association between hierarchical visual areas and the complexity levels of visual features in image feature decoding accuracy.

**Figure 4:**
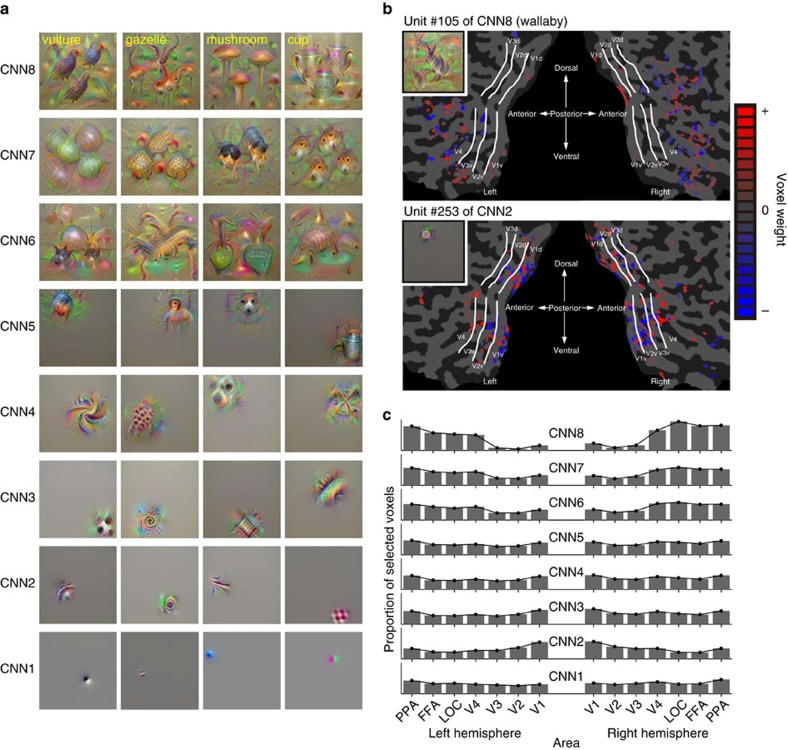
Preferred images and weight distributions for CNN layers. (**a**) Examples of preferred images for individual units (four randomly selected units) in each CNN layer. Because each unit of CNN8 corresponds to a specific category, the names of the categories for each unit are shown at the top left. (**b**) Voxel weight maps on the flattened cortical surface. Weights resulting from the decoders trained to predict feature values of a single feature unit are shown for Unit #105 of CNN8 and for Unit #253 of CNN2 (predicted from VC, Subject 3). The preferred image for each unit is indicated in the inset. (**c**) Distributions of selected voxels across individual subareas for CNN layers. Distributions of selected voxels used for prediction are shown for each CNN layer (predicted from VC, averaged across five subjects). The proportion of selected voxels for each subarea was calculated by first counting the numbers of selected voxels for individual feature units, aggregating them over ∼1,000 feature units in each layer, and then normalizing with the total voxels.

To understand more details about what each visual feature represents, we synthesized preferred images that strongly activated individual units in each CNN layer using the activation maximization technique^33^,^34^,^35^,^36^ (Fig. 4; see Supplementary Fig. 4 for more examples; see Methods section). The generated images showed gradually increasing complexity, from simple edge-detector-like representations to more complex shapes, textures or object parts and to intact objects. Because CNN6–8 are fully connected layers, position information about their preferred pattern was lost. These preferred images of individual CNN units resemble the critical features found in monkey electrophysiological studies^37^.

Analyses of voxel weights learned by the image feature decoders (trained with VC) showed differences in the spatial distribution of predictive voxels between visual feature types/layers. We used a linear regression model in which voxel weights were estimated with sparseness priors^32^, resulting in a small subset of voxels selected with non-zero weights (hence relevant for decoding). Examples of voxel sets for a CNN2 unit and a CNN8 unit are shown in Fig. 4b. While the voxels selected for predicting a CNN2 unit were distributed mainly around the lower visual areas (V1–V3), those for predicting a CNN8 unit were distributed around more anterior areas (Fig. 4b). Distributions of predictive voxels for the visual feature types/layers were consistent with the results of the image feature decoding analysis using individual ROIs (Fig. 3b): voxels in the lower/higher ROIs were more frequently selected for predicting lower/higher visual features, respectively (Fig. 4c; ANOVA, interaction between feature type/layer and ROI, *P*<0.01; see Supplementary Fig. 5 for distributions for HMAX1–3, GIST and SIFT+BoF).

**Figure 5:**
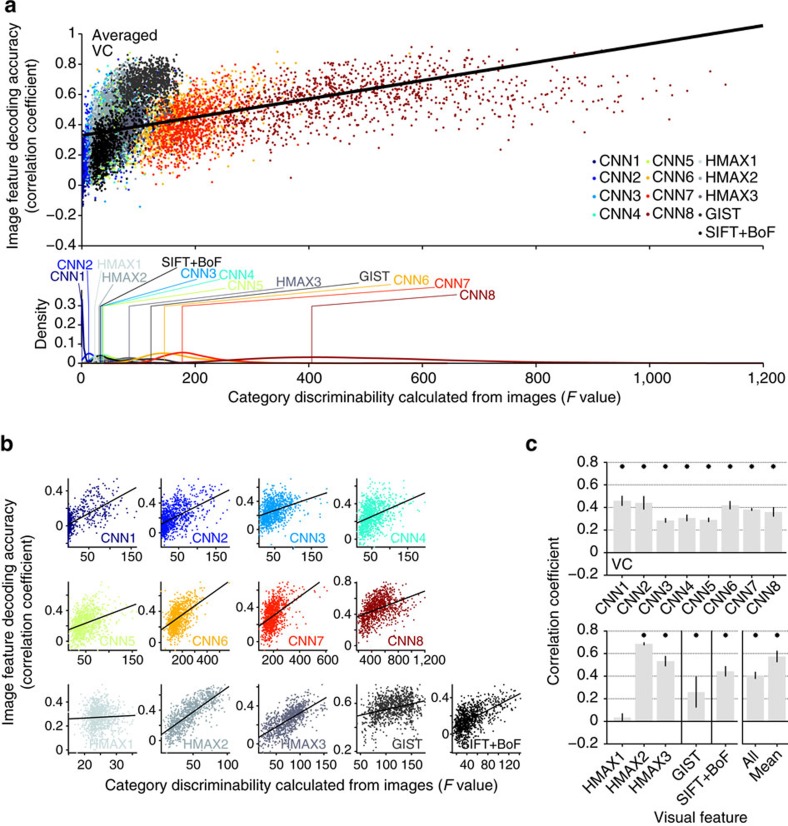
Category discriminability versus decodability of individual feature units. (**a**) Scatterplot of image feature decoding accuracy against category discriminability for all the feature types/layers (top panel, predicted from VC, averaged across five subjects for each unit), and the distributions of category discriminability for each feature type/layer (bottom panel; vertical lines denote the mode of each distribution). Each dot in the scatter plot denotes the *F*-statistic and the decoding accuracy for a feature unit (∼1,000 units). (**b**) Scatterplots for individual feature types/layers. (**c**) Correlation coefficients between category discriminability and image feature decoding accuracy (error bars, 95% CI across five subjects; asterisks, one-sided *t*-test after Fisher's *z*-transform, uncorrected *P*<0.05). The solid line in the scatterplots indicates a fitted regression line.

Additionally, we found that the image feature decoding accuracy in each unit was positively correlated with the ‘category discriminability' of each unit (Fig. 5). As an index of category discriminability, we calculated the *F*-statistic of each feature unit (a ratio of inter- and intra-category variations of feature values calculated from images in the ImageNet (15,322 categories)). The distributions of the category discriminability from each feature type/layer showed that high-level features tended to demonstrate high discriminability for both CNN and HMAX (Fig. 5a bottom). Within each feature type/layer, positive correlations between decoding accuracy and category discriminability were observed for all visual features except for HMAX1 (Fig. 5b,c). Furthermore, decoding accuracy and category discriminability were positively correlated even when feature units were combined across all feature types/layers (Fig. 5c, all), and were averaged within each feature type/layer (Fig. 5c, mean). While this analysis was performed with decoders trained on whole VC activity, this tendency was robustly reproduced in each ROI. Thus decodable feature units tended to be critical for defining object categories.

### Category-average feature decoding

In computer vision, object recognition is typically performed by matching the feature vector of an input image with a set of category-specific feature vectors, assuming that hierarchical features are progressively rendered invariant to represent object categories. To link image feature decoding with object recognition, we next tested whether the feature values from the image feature decoders (*cf.*, Fig. 3) predicted the values of category-specific feature vectors. We constructed category-specific feature vectors by averaging the feature vectors of multiple images annotated with the same object category (15,372 categories in ImageNet^31^). To evaluate the prediction accuracy in each unit, Pearson's correlation coefficients were calculated between the predicted and the category-average feature values for the series of test trials. We then averaged the correlation coefficients across the units in each feature type/layer. Evaluation with category-average features allowed us to extend the feature decoding analysis to the imagery experiment, in which subjects freely imagined about an object cued by text, and thus there were no ground truth images from which visual features could be calculated.

**Figure 6:**
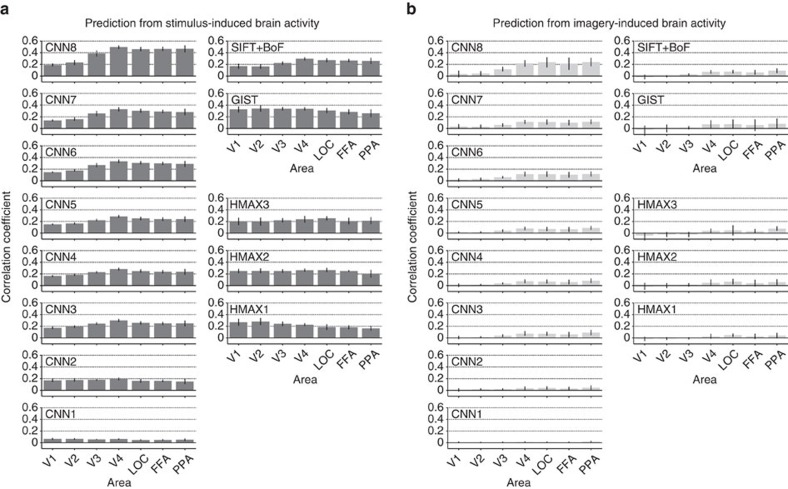
Prediction of category-average features from stimulus- and imagery-induced brain activity. (**a**) Correlation coefficients with predicted features from stimulus-induced brain activity. (**b**) Correlation coefficients with predicted features from imagery-induced brain activity. Mean correlation coefficients are shown for each feature type/layer and ROI (error bars, 95% CI across five subjects). See Supplementary Fig. 6 for the relation between category discriminability and prediction accuracy (*cf.*, Fig. 5c). Similar analyses can be performed using the decoders trained with category-average features (not image features) for training stimulus images (category-average feature decoders), showing qualitatively similar prediction results with higher accuracy for imagery-induced brain activity (Supplementary Figs 7 and 8b).

Correlation coefficients between the features decoded from stimulus- and imagery-induced brain activity and the category-average features in multiple ROIs are shown in Fig. 6 (see Supplementary Fig. 8 for distributions of correlation coefficients for individual units). Features decoded from stimulus-induced brain activity were significantly correlated with the category-average features for all feature–ROI combinations (Fig. 6a; one-sided *t*-test after Fisher's *z*-transform, uncorrected *P*<0.05). In contrast to image features (Fig. 3b), category-average features were better predicted in higher than lower ROIs for most feature types/layers. Imagery-induced brain activity showed a similar prediction pattern, but with reduced accuracies: relatively high correlations were found for mid-to-high-level CNN features using mid-to-high-level ROIs (V4, LOC, FFA and PPA) (Fig. 6b). Thus the results showed that image features decoded from stimulus- and imagery-induced brain activity were predictive of category-specific features, particularly with mid-to-high-level CNN features decoded from mid-to-high-level ROIs. Poorer prediction with other features and ROIs may be due to the lack of invariance to image attributes that were irrelevant for object recognition. The capacity of imagery-induced brain activity to predict mid-level, as well as top-level, CNN features suggests that mental imagery may recruit neural representations of visual features with intermediate complexity, which are not simply pictorial or conceptual, via progressive top–down processing.

**Figure 7:**
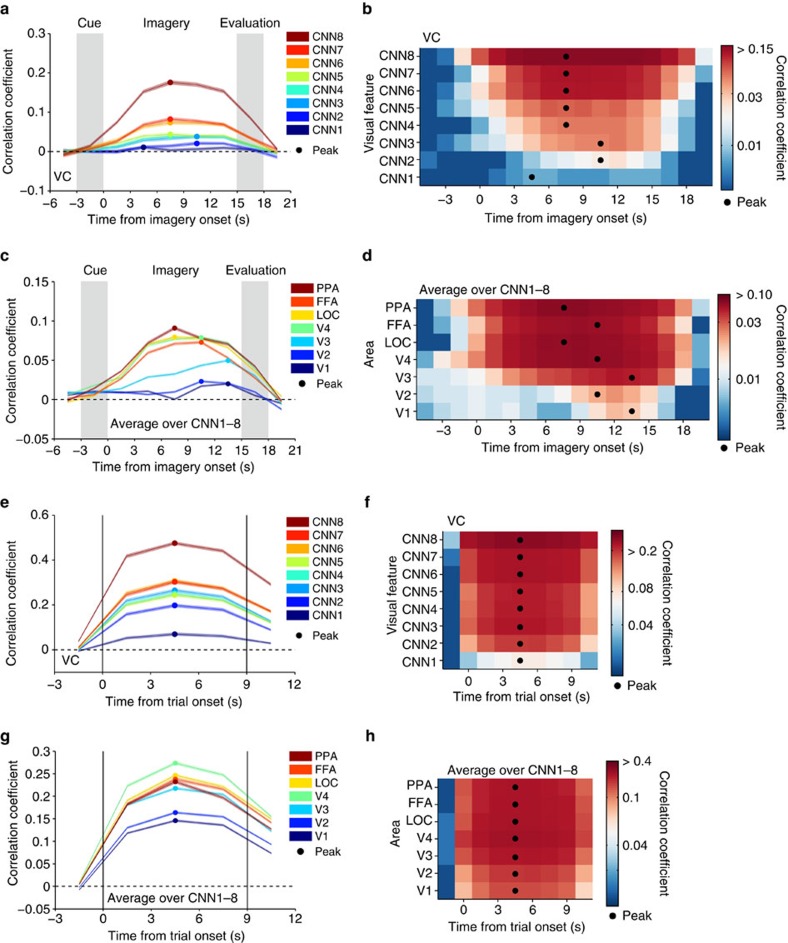
Time course of feature prediction from imagery- and stimulus-induced brain activity. At each time point/volume around the task period, correlation coefficients were calculated between the predicted and the category-average feature values for the series of test trials (averaged across five subjects; shaded areas, 95% CI across feature units; filled circles, peak timing). (**a**,**b**) Line plots and colour display for prediction from imagery-induced brain activity (VC) at different CNN layers. (**c**,**d**) Prediction from imagery-induced brain activity in individual ROIs (averaged over CNN1–8). (**e**,**f**) Prediction from stimulus-induced brain activity (VC) at different CNN layers. (**g**,**h**) Prediction from stimulus-induced brain activity in individual ROIs (average over CNN1–8). Analyses were performed on the volume-by-volume basis while colour maps are drawn by averaging time courses evaluated by every single volume or the averages of every two or three volumes, by allowing overlap for display purposes.

To find out a signature of such progressive top–down processing, we performed a time-resolved feature prediction analysis. While the results in Fig. 6 are based on the average brain activity during the entire 9-s stimulus or 15-s imagery period, the time course of prediction accuracies at each time point reveals differences between CNN layers and ROIs (Fig. 7). When the features in each CNN layer were predicted from imagery-induced brain activity in the whole VC, the peak timings for higher CNN layers tended to precede those for lower CNN layers, except CNN1, which showed poor prediction accuracy and no clear peak (Fig. 7a,b; ANOVA, interaction between time and CNN layer, *P*<0.01). Similarly, when feature prediction accuracies for all layers were averaged for each ROI, the peak timings for higher ROIs tended to precede those for lower ROIs (Fig. 7c,d; ANOVA, interaction between time and ROI, *P*<0.01; see Supplementary Fig. 9 for the time courses for each CNN layer). Such time differences across CNN layers or ROIs were not found with stimulus-induced brain activity (Fig. 7e–h). Although anecdotally, subjects reported that, in the imagery task of this study, it often took several seconds for vivid visual imagery to develop. Thus, the imagery task may have progressively activated hierarchical neural representations in a top-to-bottom manner over several seconds in concert with the vividness of imagery.

### Object category identification

**Figure 8:**
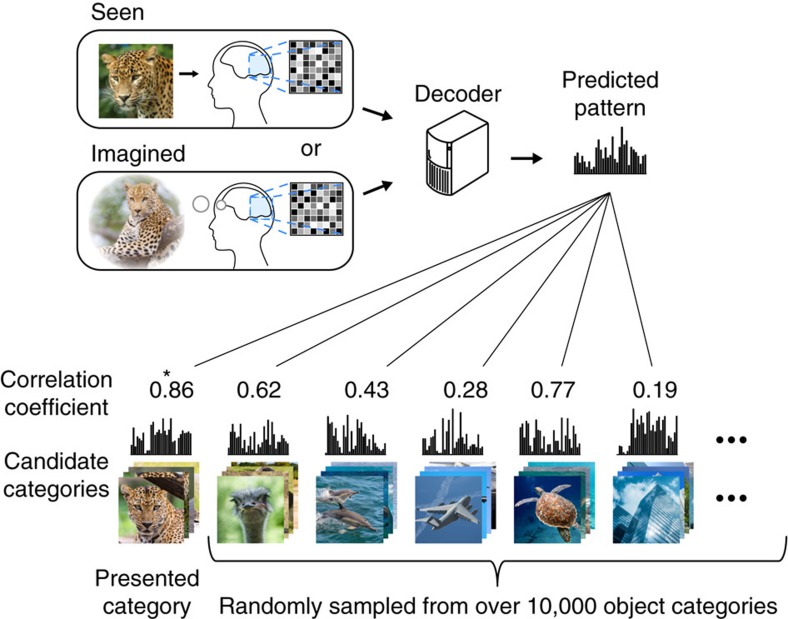
Category identification procedure. Correlation coefficients were calculated between predicted feature vectors and category-average feature vectors for categories in the candidate set, consisting of the presented or imagined category and a specified number of categories randomly selected from the ImageNet database^31^. The category with the highest correlation coefficient was selected as the predicted category (marked by a star).

We next conducted identification analysis^14^,^16^ to examine whether a predicted feature vector was useful for identifying a seen or imagined object. Because our approach is not constrained by the categories used for decoder training, we can perform identification analysis for thousands of object categories, including those not used for model training. Here the category of the seen or imagined object was identified from a variable number of candidate categories (Fig. 8). We constructed the candidate feature vector set consisting of object categories used in the test image session (and imagery experiments) and a specified number of object categories randomly selected from the 15,322 categories provided by ImageNet^31^. Given an fMRI sample, category identification was performed by selecting the category-average feature vector with the highest correlation coefficient with the predicted feature vector.

**Figure 9:**
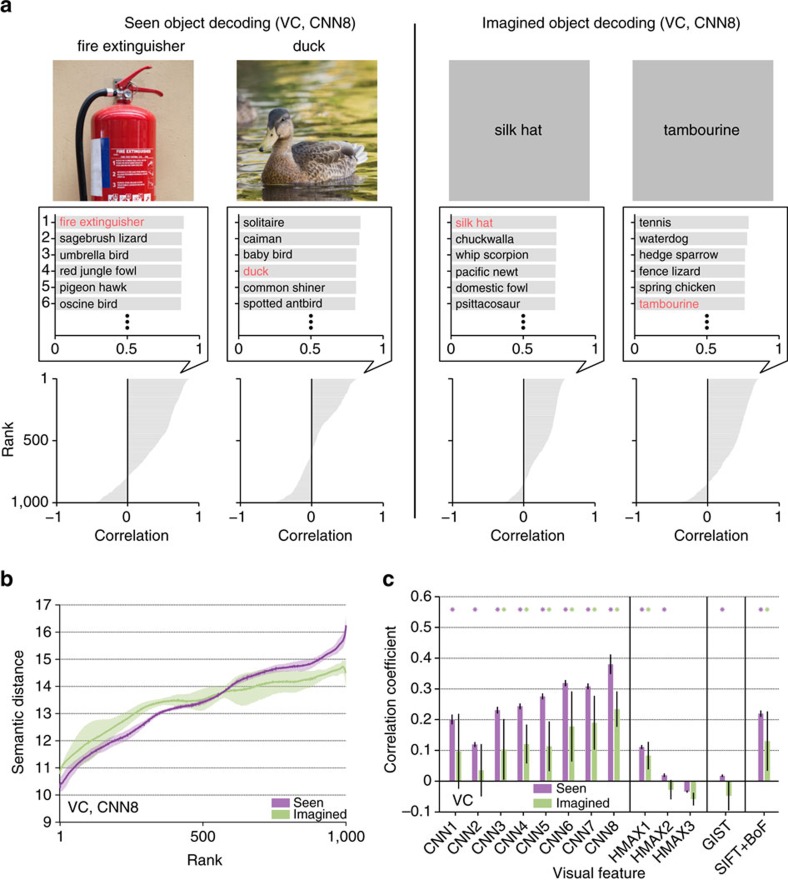
Object categories ranked by similarity to decoded feature vectors. (**a**) Object category rankings and correlation scores. Rankings of object categories are shown for two viewed objects (‘fire extinguisher' and ‘duck') and two imagined objects (‘silk hat' and ‘tambourine'; CNN8; predicted from VC; candidate sets consisting of each one of the true categories (*n*=50) and 999 randomly selected false categories). The candidate object names and correlation scores for the top six objects with the highest correlation coefficients are shown in the middle panels (correct object names indicated in red). The panels at the bottom illustrate the correlation coefficients for 1,000 object categories in descending order. (**b**) Relationship between the object category ranking and the semantic distance (CNN8; predicted from VC; shaded areas, 95% CI across five subjects). (**c**) Correlation coefficients between the rank and the semantic distance for all feature types/layers (predicted from VC). Correlation coefficients were calculated for each candidate set and target category and then averaged across 1,000 repetitions of candidate selection and 50 target categories (error bars, 95% CI across five subjects; asterisks, one-sided *t*-test after Fisher's *z*-transform, uncorrected *P*<0.05).

First, we illustrated examples with the top six categories selected from 1,000 candidates (Fig. 9a). For both seen and imagined objects, the true categories were correctly selected or highly ranked. Even when the correct categories were not assigned, the top six categories appeared to include similar or related categories (for example, the decoded feature vector for ‘duck' misidentified as another type of bird ‘solitaire').

Next, we quantitatively evaluated the relationships between rank and semantic distance with respect to the target categories and the categories ranked in each position. Semantic distance was defined by the shortest path length between the categories in the WordNet tree^38^ and was calculated between the target category and each of the 1,000 candidate categories. The 1,000 distance values were then sorted by the object category ranking (the similarity to the decoded feature vector) and averaged over 1,000 repetitions of random candidate selection and 50 target categories. The analysis showed that categories ranked in higher positions tended to show shorter semantic distances to target categories (Fig. 9b). Semantic distance was positively correlated with rank, especially for mid-to-high-level CNN layers (CNN3–8) and SIFT+BoF under both seen and imagined conditions (Fig. 9c; asterisks, one-sided *t*-test after Fisher's *z*-transform, uncorrected *P*<0.05). These results suggest that, for these feature types/layers, semantically similar, if not correct, categories can be selected with the feature vector predicted from brain activity.

**Figure 10:**
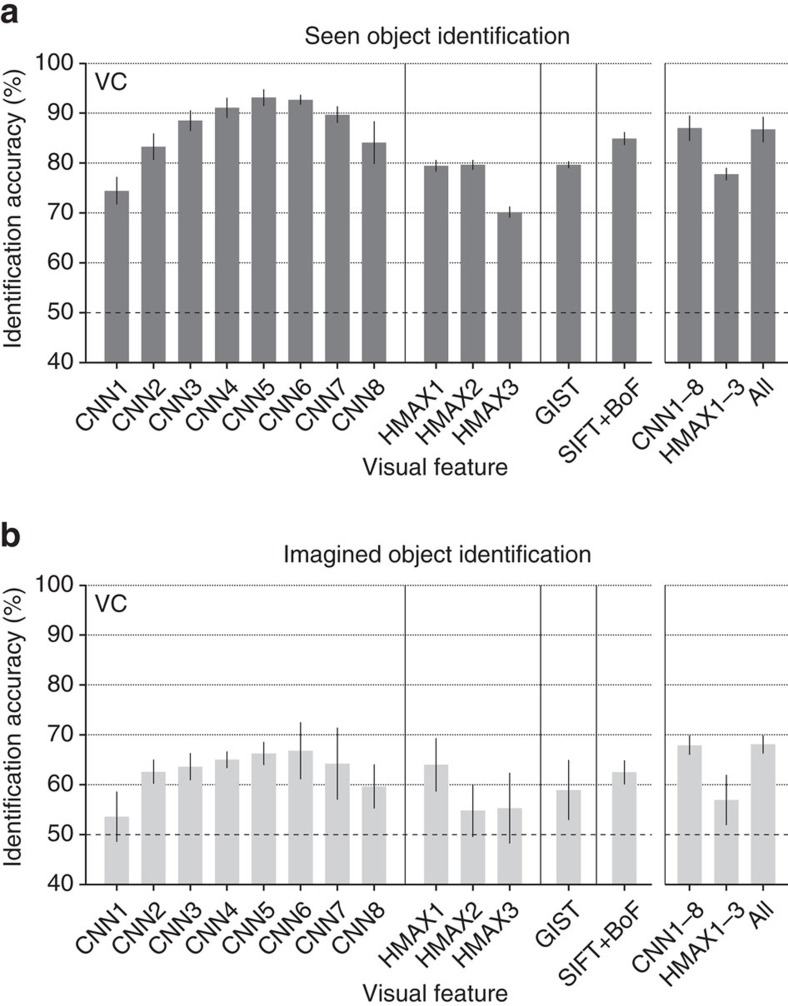
Identification accuracy. Identification was performed for all combinations of 1 of the 50 test object categories and 1 of the 15,322 candidate categories (identification from two categories; predicted from VC: error bars, 95% CI across five subjects; dashed line, chance level, 50%). (**a**) Seen object identification accuracy. (**b**) Imagined object identification accuracy. While the identification analyses here were performed with the image feature decoders trained to predict image features of presented images, it is also possible to use category-average feature decoders trained with category-average features for training stimulus images. See Supplementary Figs 12 and 13 for the results of the category-average feature decoders.

For quantitative evaluation, we assessed identification accuracy of seen and imagined objects when the number of candidate sets was two (Fig. 10a,b; see Supplementary Fig. 10 for identification accuracy as a function of the number of average samples). This analysis was performed with feature vectors for individual feature types/layers and with concatenated feature vectors for CNN1–8 (8,000 units), HMAX1–3 (3,000 units) and all 13 feature types/layers (13,024 units).

The analysis revealed that both seen and imagined objects were successfully identified with statistically significant accuracy for most of the feature types/layers (one-sided *t*-test, uncorrected *P*<0.05 except for CNN1, HMAX2 and HMAX3 under the imagery condition) with highest accuracy around mid-level features (CNN5–6). Furthermore, when the same analysis was performed for each ROI, above-chance accuracy was achieved for most feature–ROI combinations (Supplementary Fig. 11). Intriguingly, mid-level features decoded from higher ROIs were most useful for identifying both seen and imagined object categories.

Additional analyses revealed that the pairs with larger semantic distances (WordNet's path length) tended to have higher identification accuracies (Supplementary Fig. 14), consistent with the relationships between the identified rank and semantic distance (Fig. 9c). Because we used a pretrained CNN model, the 1,000 categories used in the model accidentally included 20 of the test categories in our study. However, the identification accuracy with the other 30 non-overlapping categories alone was qualitatively similar to the main results (Supplementary Fig. 15).

## Discussion

We have shown that via hierarchical visual feature representation, arbitrary object categories seen and imagined by subjects can be predicted from fMRI signals in the human VC. The trained decoders successfully predicted the feature values of the presented images. Higher-/lower-order visual features tended to be better predicted from fMRI signals in higher/lower cortical areas, respectively. Further, decoders trained to predict feature vectors of presented images can be used to predict those of both seen and imagined object categories, enabling the identification of seen and imagined object categories despite not using the same categories for decoder training. Interestingly, mid-level features were most useful for identifying object categories, suggesting the significant contributions of mid-level features to construct discriminative representations for object categories. Thus, our results demonstrate that a decoding model trained on a limited set of object categories generalizes to decode arbitrary object categories, providing a proof of concept for generic object decoding. This framework is also known as the ‘zero-data learning' or ‘zero-shot learning' in the machine-learning field^39^, in which a model must generalize to classes with no training data. Moreover, successful predictions of category-average features at mid-to-high-level CNN layers and object category identification from imagery-induced brain activity in mid-to-high-level ROIs would suggest that mental imagery may recruit neural representations of visual features with intermediate complexity, which are elicited in visual perception, via progressive top–down processing.

Our analyses demonstrated that visual features extracted by computational models were successfully predicted from brain activity patterns (Fig. 3). The analysis revealed a hierarchical correspondence between cortical hierarchy and the levels of visual feature representations. These results were consistent with previous demonstrations of high representational similarity between the top layer of a CNN and visual cortical activity in the inferior temporal cortex of humans^27^,^28^ and non-human primates^25^,^26^,^27^. Moreover, several previous studies reported that features from the middle layer of a hierarchical neural network were able to accurately predict V4 brain activity^25^,^26^. In addition, a previous study reported an explicit gradient for feature complexity in visual cortical hierarchy using an encoding approach^28^. Our results are consistent with these findings, showing a homology between the hierarchies of individual areas from lower to HVC and the CNN layers using a decoding approach. Thus our results support the idea that CNN models can provide a good proxy for the hierarchical feed-forward visual system for object recognition.

In our analyses, we selected 1,000 feature units to reduce computational cost, as some layers of the CNN (CNN1–7) originally contained >100,000 units. However, it is possible that the selection of the 1,000 units biased the results. To test this possibility, we repeated the same identification analysis by resampling units from the original feature populations and repeatedly changing the number of units from 10 to 1,000 (Supplementary Fig. 16). The analysis demonstrated that the identification accuracies of most feature types/layers were almost saturated when several hundred units were used, and qualitatively similar results to the main results were obtained for different numbers of units.

Our results may be relevant to the long-standing debate regarding whether mental imagery is symbolic (language-like) or depictive (picture-like)^40^,^41^. In our analysis, the decoders trained on brain activity induced by visual stimuli were able to generalize to predict the category-average feature vectors of not only seen but also imagined object categories (Fig. 5), enabling the identification of imagined object categories using the feature vector decoded from brain activity during imagery (Fig. 10b). Previous studies reported that the common neural representations are used during perception and mental imagery for low-level image properties^3^,^4^,^5^,^17^, including information about orientation, spatial frequency and retinotopic location, as well as for high-level semantic and conceptual representations^8^,^9^,^10^,^12^, depending on the tasks performed by subjects. Our analyses further revealed progressive recruitment of multiple levels of hierarchical visual features, including features with intermediate complexity that go beyond low-level image properties and bridge the gap between pictorial and conceptual representations (Fig. 7). Our analyses quantify the top–down effects of hierarchical visual cortical activity during mental imagery, suggesting that feature-level representations elicited in visual perception were recruited during mental object imagery in a graded manner. Taken together, these results reveal the nature of mental imagery as a type of top–down perception.

We extended the modular decoding approach previously proposed in our visual image reconstruction study^16^ to high-level object vision using visual features with multiple levels of complexity (Fig. 4a). Since images depicting the same kinds of objects do not necessarily have pixel-wise similarity, complex and invariant features would appear to be suitable for object identification. However, a simulation study suggested that intermediate level rather than highly complicated visual features are more useful for object discrimination^42^. Our additional analysis with true feature vectors calculated from stimulus images (with no prediction errors; equivalent to generic object recognition in machine vision) also showed a slightly poorer identification with CNN8 than with CNN7 (Supplementary Fig. 17). Consistent with these observations, our results from decoded feature vectors showed the highest identification accuracy with mid-level rather than top-level CNN features. These results suggest the suitability of mid-level, or generic, features for discriminative object representation, possibly consistent with electrophysiological studies of monkey inferior temporal cortex^37^,^43^.

Our approach is relevant to a previous study that focussed on semantic feature representations to establish relations between word meanings and brain activity^44^. The study demonstrated decoding of arbitrary nouns thought by subjects using encoding models and statistics of word co-occurrence. Our work differs from that attempt in that we employed computational models that produced visual feature representations from images to establish the relations between brain activity in VC and object categories, making it possible to address how hierarchical visual feature representations associated with visual objects are recruited during mental imagery. Furthermore, in the experiment of the previous study^44^, subjects were presented with both line drawings and noun labels of concrete objects and were instructed to think about the properties of the target object. In contrast, in our image presentation experiment, visual images were presented alone with no cognitive task other than the one-back repetition task to maintain subjects' attention, and in the imagery experiment, subjects were only required to imagine visual images of the target category without any additional tasks. Our results demonstrated that imagining about object images is sufficient to achieve generic decoding of imagined object categories utilizing the commonality of feature-level representations between perception and imagery.

In the present study, we used decoding approach instead of the representational similarity analysis^27^,^45^,^46^,^47^,^48^ or encoding approaches^14^,^15^,^17^,^25^,^26^,^28^ to link brain activity and visual features (see Supplementary Figs 18–20 for analyses of the encoding approach). While the representational similarity analysis and encoding approaches can evaluate mass characteristics of each visual features (for example, a specific layer of the CNN) associated with brain activity, our decoding approach can characterize individual feature units in terms of decodability from distributed brain activity patterns. As previously demonstrated, computational models with a high representational similarity to brain activity patterns in the inferior temporal cortex showed better categorization accuracy^27^. Thus it may be possible to use the prediction accuracy of individual units as a guide to find effective units for better object recognition performance in machine vision. Indeed, our analyses revealed that the feature units better predicted from brain activity showed higher category discriminability (Fig. 5). This finding is consistent with the conclusions of previous studies^25^,^26^,^27^ and supports the notion that feature decoding accuracy may be a suitable guide for selecting more useful features for object recognition.

Our identification analyses revealed that highly ranked candidate categories tended to be semantically similar to the target categories for mid-to-high-level CNN layers and SIFT+BoF (Fig. 9b,c). Therefore, even when identification was incorrect, we were able to predict semantically similar categories to the target category. However, this tendency was not observed with HMAX or GIST. Because GIST captures low-level image properties^24^,^29^, it is not surprising that rank and the semantic distance were not correlated. Conversely, HMAX was designed to model the higher-level visual system, but did not show a positive correlation between rank and semantic distance. This may suggest that HMAX cannot capture the semantics of objects, which is consistent with the indications of several previous studies^27^,^45^,^46^,^47^,^48^.

Our approach allows the decoding of arbitrary object categories not limited to those used for decoder training. This new method has a range of potential practical applications, particularly in situations where what kind of objects should be decoded is unknown. Because our approach can decode imagined categories, it may also be possible to decode the contents of dreaming or daydreaming^13^. Reading the contents of such spontaneously generated thinking may be beneficial for understanding the functions of such cognitive phenomena. Achieving this requires distinguishing the conceivable differences in neural representation between volitional and spontaneous mental imagery. This formulates a challenging problem in future research. In addition, our approach may provide a basis for a brain-based information retrieval system by translating brain activity into words or concepts. Using the outputs of decoders, our approach may create a query for an information retrieval system based on brain activity.

The framework of directly predicting visual features from brain activity may be utilized for applications developed with deep neural networks. Recent advances have enabled image reconstruction^49^ and description generation^50^ from feature patterns obtained by processing images through CNN. Combining these technologies with brain decoding may extend previous reconstruction study^16^ and the present work to produce richer outputs. Our results demonstrating the predictability of CNN features from the brain may then open the possibility to develop new technology for brain machine interface by combining brain decoding and deep neural networks.

## Methods

### Subjects

Five healthy subjects (one female and four males, aged between 23 and 38 years) with normal or corrected-to-normal vision participated in the experiments. Rather than using statistical methods to determine the sample size, the sample size was chosen to match previous fMRI studies with similar behavioral protocols. All subjects had considerable experience participating in fMRI experiments, and were highly trained. All subjects provided written informed consent for participation in the experiments, and the study protocol was approved by the Ethics Committee of ATR.

### Visual images

Images were collected from an online image database ImageNet^31^ (2011, fall release), an image database where images are grouped according to the hierarchy in WordNet^38^. We selected 200 representative object categories (synsets) as stimuli in the visual image presentation experiment. After excluding images with a width or height <100 pixels or aspect ratio >1.5 or <2/3, all remaining images in ImageNet were cropped to the centre. For copyright reasons, the images in Figs 1, 2, 3, 8 and 9 are not the actual images from ImageNet used in our experiments. The original images are replaced with images with similar contents for display purposes.

### Experimental design

We conducted two types of experiments: an image presentation experiment, and an imagery experiment. All visual stimuli were rear-projected onto a screen in an fMRI scanner bore using a luminance-calibrated liquid crystal display projector. Data from each subject were collected over multiple scanning sessions spanning approximately 2 months. On each experiment day, one consecutive session was conducted for a maximum of 2 hours. Subjects were given adequate time for rest between runs (every 3–10 min) and were allowed to take a break or stop the experiment at any time.

The image presentation experiment consisted of two distinct types of sessions: training image sessions and test image sessions, each of which consisted of 24 and 35 separate runs (9 min 54 s for each run), respectively. Each run contained 55 stimulus blocks consisting of 50 blocks with different images and five randomly interspersed repetition blocks where the same image as in the previous block was presented. In each stimulus block, an image (12 × 12 degrees of visual angle) was flashed at 2 Hz for 9 s. Images were presented at the centre of the display with a central fixation spot. The colour of the fixation spot changed from white to red for 0.5 s before each stimulus block began to indicate the onset of the block. Extra 33- and 6-s rest periods were added to the beginning and end of each run, respectively. Subjects maintained steady fixation throughout each run and performed a one-back repetition detection task on the images, responding with a button press for each repetition to maintain their attention on the presented images (mean task performance across five subjects; sensitivity=0.930; specificity=0.995). In the training image session, a total of 1,200 images from 150 object categories (8 images from each category) were each presented only once. In the test image session, a total of 50 images from 50 object categories (1 image from each category) were presented 35 times each. Importantly, the categories in the test image session were not used in the training image session. The presentation order of the categories was randomized across runs.

In the imagery experiment, subjects were required to visually imagine images from 1 of the 50 categories that were presented in the test image session of the image presentation experiment. Prior to the experiment, 50 image exemplars from each category were exposed to train the correspondence between object names and the visual images specified by the names. The imagery experiment consisted of 20 separate runs and each run contained 25 imagery blocks (10 min 39 s for each run). Each imagery block consisted of a 3-s cue period, a 15-s imagery period, a 3-s evaluation period and a 3-s rest period. Extra 33- and 6-s rest periods were added to the beginning and end of each run, respectively. During the rest periods, a white fixation spot was presented at the centre of the display. The colour of the fixation spot changed from white to red for 0.5 s to indicate the onset of the blocks from 0.8 s before each cue period began. During the cue period, words describing the names of the 50 categories presented in the test image session were visually presented around the centre of the display (1 target and 49 distractors). The position of each word was randomly changed across blocks to avoid contamination of cue-specific effects on the fMRI response during imagery periods. The word corresponding to the category to be imagined was presented in red (target) and the other words were presented in black (distractors). The onset and end of the imagery periods were signaled by beep sounds. Subjects were required to start imagining as many object images pertaining to the category described by the red word as possible, and were instructed to keep their eyes closed from the first beep until the second beep. After the second beep, the word corresponding to the target category was presented to allow the subjects evaluate the vividness of their mental imagery on a five-point scale (very vivid, fairly vivid, rather vivid, not vivid, cannot recognize the target) by a button press. The 25 categories in each run were pseudo-randomly selected from 50 categories such that the two consecutive runs contained all 50 categories.

### Retinotopy experiment

The retinotopy experiment followed the conventional protocol^51^,^52^ using a rotating wedge and an expanding ring of a flickering checkerboard. The data were used to delineate the borders between each visual cortical area and to identify the retinotopic map (V1–V4) on the flattened cortical surfaces of individual subjects.

### Localizer experiment

We performed functional localizer experiments to identify the LOC, FFA and PPA for each individual subject^53^,^54^,^55^. The localizer experiment consisted of 4–8 runs and each run contained 16 stimulus blocks. In this experiment, intact or scrambled images (12 × 12 degrees of visual angle) from face, object, house and scene categories were presented at the centre of the screen. Each of the eight stimulus types (four categories × two conditions) was presented twice per run. Each stimulus block consisted of a 15-s intact or scrambled stimulus presentation. The intact and scrambled stimulus blocks were presented successively (the order of the intact and scrambled stimulus blocks was random), followed by a 15-s rest period consisting of a uniform grey background. Extra 33- and 6-s rest periods were added to the beginning and end of each run, respectively. In each stimulus block, 20 different images of the same type were presented for 0.3 s, followed by an intervening blank screen of 0.4 s.

### MRI acquisition

fMRI data were collected using 3.0-Tesla Siemens MAGNETOM Trio A Tim scanner located at the ATR Brain Activity Imaging Center. An interleaved T2*-weighted gradient-EPI (echo-planar imaging) scan was performed to acquire functional images covering the entire brain (image presentation, imagery and localizer experiments: repetition time (TR), 3,000 ms; echo time (TE), 30 ms; flip angle, 80 deg; field of view (FOV), 192 × 192 mm^2^; voxel size, 3 × 3 × 3 mm^3^; slice gap, 0 mm; number of slices, 50) or the entire occipital lobe (retinotopy experiment: TR, 2,000 ms; TE, 30 ms; flip angle, 80 deg; FOV, 192 × 192 mm^2^; voxel size, 3 × 3 × 3 mm^3^; slice gap, 0 mm; number of slices, 30). T2-weighted turbo spin echo images were scanned to acquire high-resolution anatomical images of the same slices used for the EPI (image presentation, imagery and localizer experiments: TR, 7,020 ms; TE, 69 ms; flip angle, 160 deg; FOV, 192 × 192 mm^2^; voxel size, 0.75 × 0.75 × 3.0 mm^3^; retinotopy experiment: TR, 6,000 ms; TE, 57 ms; flip angle, 160 deg; FOV, 192 × 192 mm^2^; voxel size, 0.75 × 0.75 × 3.0 mm^3^). T1-weighted magnetization-prepared rapid acquisition gradient-echo fine-structural images of the entire head were also acquired (TR, 2,250 ms; TE, 3.06 ms; TI, 900 ms; flip angle, 9 deg, FOV, 256 × 256 mm^2^; voxel size, 1.0 × 1.0 × 1.0 mm^3^).

### MRI data preprocessing

The first 9-s scans for experiments with TR=3 s (image presentation, imagery and localizer experiments) and 8-s scans for experiments with TR=2 s (retinotopy experiment) of each run were discarded to avoid MRI scanner instability. The acquired fMRI data underwent three-dimensional motion correction using SPM5 (http://www.fil.ion.ucl.ac.uk/spm). The data were then coregistered to the within-session high-resolution anatomical image of the same slices used for EPI and subsequently to the whole-head high-resolution anatomical image. The coregistered data were then reinterpolated by 3 × 3 × 3 mm^3^ voxels.

For the data from the image presentation experiment and imagery experiment, after within-run linear trend removal, voxel amplitudes were normalized relative to the mean amplitude of the entire time course within each run. The normalized voxel amplitudes from each experiment were then averaged within each 9-s stimulus block (three volumes; image presentation experiment) or within each 15-s imagery period (five volumes; imagery experiment), respectively (unless otherwise stated) after shifting the data by 3 s (one volume) to compensate for haemodynamic delays.

### ROI selection

V1–V4 were delineated by the standard retinotopy experiment^51^,^52^. The retinotopy experiment data were transformed to Talairach coordinates and the visual cortical borders were delineated on the flattened cortical surfaces using BrainVoyager QX (http://www.brainvoyager.com). The voxel coordinates around the grey–white matter boundary in V1–V4 were identified and transformed back into the original coordinates of the EPI images. The voxels from V1 to V3 were combined, and defined as the ‘LVC'. The LOC, FFA and PPA were identified using conventional functional localizers^53^,^54^,^55^. The localizer experiment data were analysed using SPM5. The voxels showing significantly higher responses to objects, faces or scenes than for scrambled images (two-sided *t*-test, uncorrected *P*<0.05 or 0.01) were identified and defined as LOC, FFA and PPA, respectively. A contiguous region covering LOC, FFA and PPA was manually delineated on the flattened cortical surfaces, and the region was defined as the ‘HVC'. Voxels overlapping with LVC were excluded from the HVC. Voxels from V1 to V4 and the HVC were combined to define the ‘VC'. In the regression analysis, voxels showing the highest correlation coefficient with the target variable in the training image session were selected to predict each feature (with a maximum of 500 voxels for V1–V4, LOC, FFA and PPA; 1,000 voxels for LVC, HVC and VC).

### Visual features

We used four types of computational models: a CNN^20^, HMAX^21^,^22^,^23^, GIST^24^ and SIFT^18^ combined with the ‘BoF'^16^ to construct visual features from images. The features with a model-training phase (HMAX and SIFT+BoF) used 1,000 images belonging to the categories used in the training image session (150 categories) for training. Each model is further described in the following subsections.

### Convolutional neural network

We used the MatConvNet implementation (http://www.vlfeat.org/matconvnet/) of the CNN model^20^, which was trained with images in ImageNet^31^ to classify 1,000 object categories. The CNN consisted of five convolutional layers and three fully connected layers. We randomly selected 1,000 units in each of the first to seventh layers and used all 1,000 units in the eighth layer. We represented each image by a vector of those units' outputs and named them CNN1–CNN8, respectively.

### HMAX

HMAX^21^,^22^,^23^ is a hierarchical model that extends the simple and complex cells described by Hubel and Wiesel^56^,^57^ and computed features through hierarchical layers. These layers consist of an image layer and six subsequent layers (S1, C1, S2, C2, S3 and C3), which are built from the previous layers by alternating template matching and max operations. In the calculations at each layer, we employed the same parameters as in a previous study^22^, except that the number of features in layers C2 and C3 was set to 1,000. We represented each image by a vector of the three types of HMAX features, which consisted of 1,000 randomly selected outputs of units in layers S1, S2 and C2, and all 1,000 outputs in layer C3. We defined these outputs as HMAX1, HMAX2 and HMAX3, respectively.

### GIST

GIST is a model developed for the computer-aided scene categorization task^24^. To compute GIST, an image was first converted to grey–scale and resized to have a maximum width of 256 pixels. Next, the image was filtered using a set of Gabor filters (16 orientations, 4 scales). After that, the filtered images were segmented by a 4 × 4 grid (16 blocks), and then the filtered outputs within each block were averaged to extract 16 responses for each filter. The responses from multiple filters were concatenated to create a 1,024-dimensional feature vector for each image (16 (orientations) × 4 (scales) × 16 (blocks)=1,024).

### SIFT with BoF (SIFT+BoF)

The visual features using the SIFT with the BoF approach were calculated from SIFT descriptors. We computed SIFT descriptors from the images using the VLFeat^58^ implementation of dense SIFT. In the BoF approach, each component of the feature vector (visualwords) is created by vector-quantizing extracted descriptors. Using ∼1,000,000 SIFT descriptors calculated from an independent training image set, we performed k-means clustering to create a set of 1,000 visualwords. The SIFT descriptors extracted from each image were quantized into visualwords using the nearest cluster centre, and the frequency of each visualword was calculated to create a BoF histogram for each image. Finally, all of the histograms obtained through the above processing underwent L-1 normalization to become unit norm vectors. Consequently, features from SIFT with the BoF approach are invariant to image scaling, translation and rotation and are partially invariant to illumination changes and affine or three-dimensional projection.

### Visual feature decoding

We constructed decoding models to predict the visual feature vectors of seen objects from fMRI activity using a linear regression function. Here we used SLR (http://www.cns.atr.jp/cbi/sparse_estimation/index.html)^32^ that can automatically select the important features for prediction. Sparse estimation is known to perform well when the dimensionality of the explanatory variable is high, as is the case with fMRI data^59^.

Given an fMRI sample 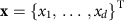 consisting of the activity of *d* voxels as input, the regression function can be expressed by


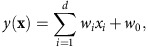


where *x*_*i*_ is a scalar value specifying the fMRI amplitude of the voxel *i*, *w*_*i*_ is the weight of voxel *i* and *w*_0_ is the bias. For simplicity, the bias *w*_0_ is absorbed into the weight vector such that 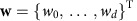. The dummy variable *x*_0_=1 is introduced into the data such that 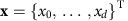. Using this function, we modeled the *l*th component of each visual feature vector as a target variable *t*_*l*_ (*l*∈{1,…, *L*}) that is explained by the regression function *y*(**x**) with additive Gaussian noise as described by


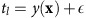


where *∈* is a zero mean Gaussian random variable with noise precision *β*.

Given a training data set, SLR computes the weights for the regression function such that the regression function optimizes an objective function. To construct the objective function, we first express the likelihood function by


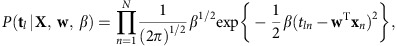


where *N* is the number of samples and **X** is an *N* × (*d*+1) fMRI data matrix whose *n*th row is the *d*+one-dimensional vector **x**_*n*_, and 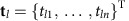 are the samples of a component of the visual feature vector.

We performed Bayesian parameter estimation and adopted the automatic relevance determination prior^32^ to introduce sparsity into the weight estimation. We considered the estimation of the weight parameter **w** given the training data sets {**X**, **t**_*l*_}. We assumed a Gaussian distribution prior for the weights **w** and non-informative priors for the weight precision parameters 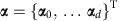 and the noise precision parameter *β*, which are described as


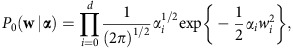



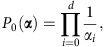



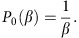


In the Bayesian framework, we considered the joint probability distribution of all the estimated parameters, and the weights can be estimated by evaluating the following joint posterior probability of **w**:


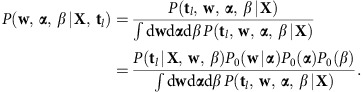


Given that the evaluation of the joint posterior probability 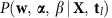 is analytically intractable, we approximated it using the variational Bayesian method^32^,^60^,^61^. While the results shown in the main figures are based on this automatic relevance determination model, we obtained qualitatively similar results using other regression models (Supplementary Figs 21 and 22).

We trained linear regression models that predict feature vectors of individual feature types/layers for seen object categories given fMRI samples in the training image session. For test data sets, fMRI samples corresponding to the same categories (35 samples in the test image session, 10 samples in the imagery experiment) were averaged across trials to increase the signal-to-noise ratio of the fMRI signals. Using the learned models, we predicted feature vectors of seen/imagined objects from averaged fMRI samples to construct one predicted feature vector for each of the 50 test categories.

### Synthesizing preferred images using activation maximization

We used the activation maximization method to generate preferred images for individual units in each CNN layer^33^,^34^,^35^,^36^. Synthesizing preferred images starts from a random image and optimizes the image to maximally activate a target CNN unit by iteratively calculating how the image should be changed via backpropagation. This analysis was implemented using custom software written in MATLAB based on Python codes provided in a series of blog posts (Mordvintsev, A., Olah, C., Tyka, M., DeepDream—a code example for visualizing Neural Networks, https://github.com/google/deepdream, 2015; Øygard, A. M.,Visualizing GoogLeNet Classes, https://github.com/auduno/deepdraw, 2015).

### Identification analysis

In the identification analyses, seen/imagined object categories were identified using the visual feature vectors decoded from fMRI signals. Prior to the identification analysis, visual feature vectors were computed for all of the preprocessed images in all of the categories (15,372 categories in ImageNet^31^) except for those used in the fMRI experiments and their hypernym/hyponym categories and those used for visual feature model training (HMAX and SIFT+BoF). The visual feature vectors of individual images were averaged within each category to create category-average feature vectors for all of the categories to form the candidate set. We computed Pearson's correlation coefficients between the decoded and the category-average feature vectors in the candidate sets. To quantify accuracy, we created candidate sets consisting of the seen/imagined categories and the specified number of randomly selected categories. None of the categories in the candidate set were used for decoder training. Given a decoded feature vector, category identification was conducted by selecting the category with the highest correlation coefficient among the candidate sets.

### Statistics

In the main analysis, we used *t*-tests to examine whether the mean of the correlation coefficients and the mean of the identification accuracies across subjects significantly exceeded the chance level (0 for correlation coefficient, and 50% for identification accuracy). For correlation coefficients, Fisher's *z*-transform was applied before the statistical tests. Before every *t*-test, we performed the Shapiro–Wilk test to check normality, and we confirmed that the null hypothesis that the data that came from a normal distribution was not rejected for all cases (*P*>0.01).

### Data and code availability

The experimental data and codes that support the findings of this study are available from our repository: https://github.com/KamitaniLab/GenericObjectDecoding.

## Additional information

**How to cite this article:** Horikawa, T. & Kamitani, Y. Generic decoding of seen and imagined objects using hierarchical visual features. *Nat. Commun.* **8,** 15037 doi: 10.1038/ncomms15037 (2017).

**Publisher's note:** Springer Nature remains neutral with regard to jurisdictional claims in published maps and institutional affiliations.

## Supplementary Material

Supplementary Information Supplementary Figures and Supplementary References
