# Supplementary material for: Generic decoding of seen and imagined objects using hierarchical visual features
Source: Nat Commun. 2017 May 22;8:15037. doi: 10.1038/ncomms15037 (PMC5458127; doi:10.1038/ncomms15037)
Supplement: Supplementary Information — Supplementary Figures and Supplementary References [file ncomms15037-s1.pdf]

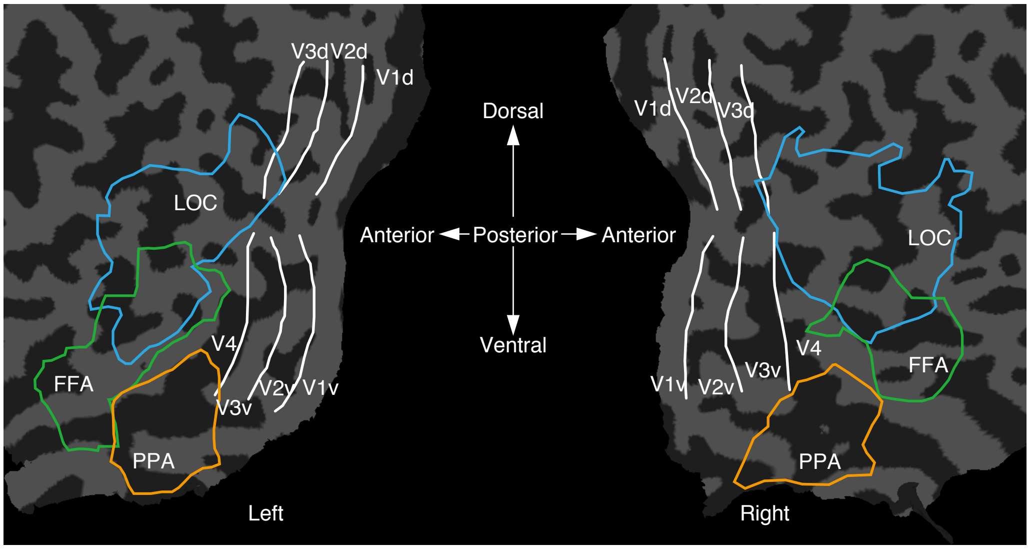

Supplementary Figure 1 | Definitions of ROIs on flattened cortex. The individual ROIs of Subject 2 are shown on the flattened cortex. A contiguous region covering the LOC, FFA, and PPA was manually delineated on the flattened cortical surface, and the region was defined as the "higher visual cortex" (HVC). Voxels overlapping with the "lower visual cortex" (LVC, V1–V3) were excluded from the ROI for the HVC. For individual ROIs voxels near the area border were included in both ROIs.

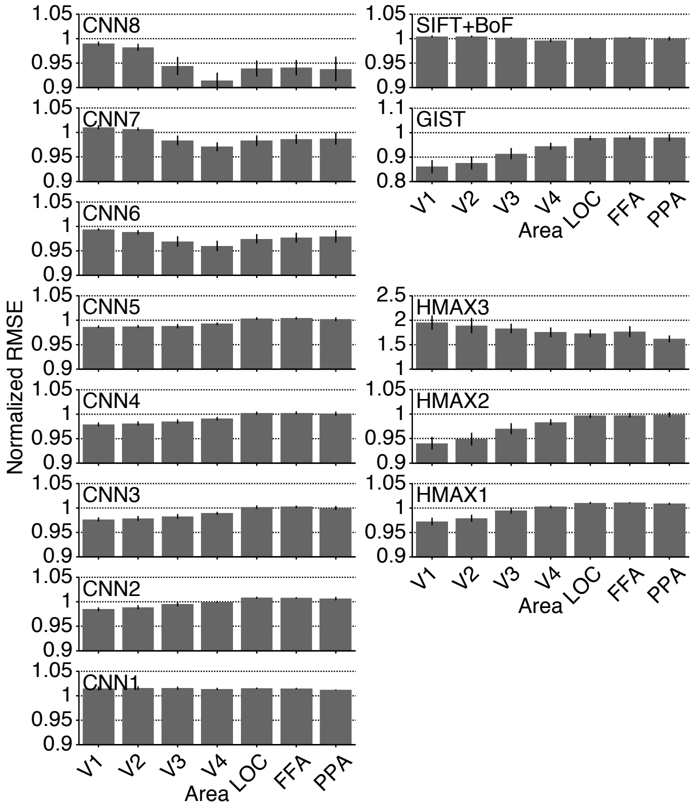

Supplementary Figure 2 | Image feature decoding accuracy evaluated by normalized root mean square error. For each feature unit, the root mean square error (RMSE) between true and predicted values were calculated over 50 test categories, and then normalized by the standard deviation of the true values. The normalized RMSE (nRMSE) was averaged for each combination of feature types/layers and ROIs (error bars, 95% CI across five subjects). The range of the horizontal axis was changed for each visual feature type/layer for display purposes. This analysis replicated a general trend observed in the results based on correlation coefficients (Fig. 3b), showing that higher-order visual features tended to be better predicted from fMRI signals in higher rather than lower ROIs, and that lower-order visual features tended to be better predicted from fMRI signals in lower rather than higher ROIs (ANOVA, interaction between visual feature type/layer and ROI, $P <$ 0.01). However, nRMSE showed a different pattern of accuracy from correlation coefficients when compared across feature types/layers. For example, HMAX3 showed the worst accuracy in the nRMSE analysis for all ROIs, although it attained a higher accuracy than several CNN features and SIFT+BoF in the correlation analysis.

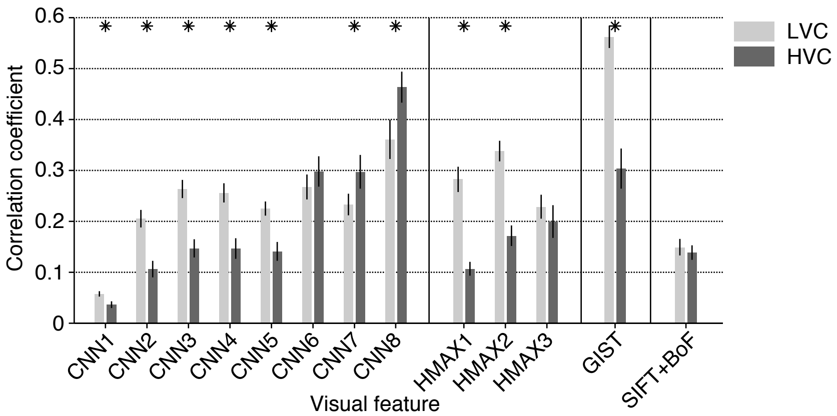

Supplementary Figure 3 | Image feature decoding accuracy obtained by decoders trained with brain activity from lower and higher ROIs. The image feature decoding accuracy obtained from decoders trained on brain activity patterns from the LVC and HVC are shown (error bars, 95% CI across five subjects). The analyses showed that the decoders trained on LVC activity outperformed those trained on HVC activity in CNN1–5, HMAX1 and 2, and GIST, while the opposite was observed in CNN7 and CNN8 (asterisk, two-sided *t*-test, uncorrected $P < 0.01$; ANOVA, interaction between visual feature type/layer and ROI, $P < 0.01$, for both of the CNN feature set and the HMAX feature set). The difference in decoding accuracy between decoders of the LVC and HVC did not reach statistical significance in CNN6, HMAX3, or SIFT+BoF (two-sided *t*-test, uncorrected $P > 0.01$). These results characterized the visual feature types/layers with respect to the levels of visual cortical hierarchy. Before the *t*-test, we performed an *F*-test to check the equality of variances between the results from the LVC and HVC. The results confirmed that the null hypothesis that the data for the LVC and HVC have the same variance was not rejected for all feature types/layers ($P > 0.05$).

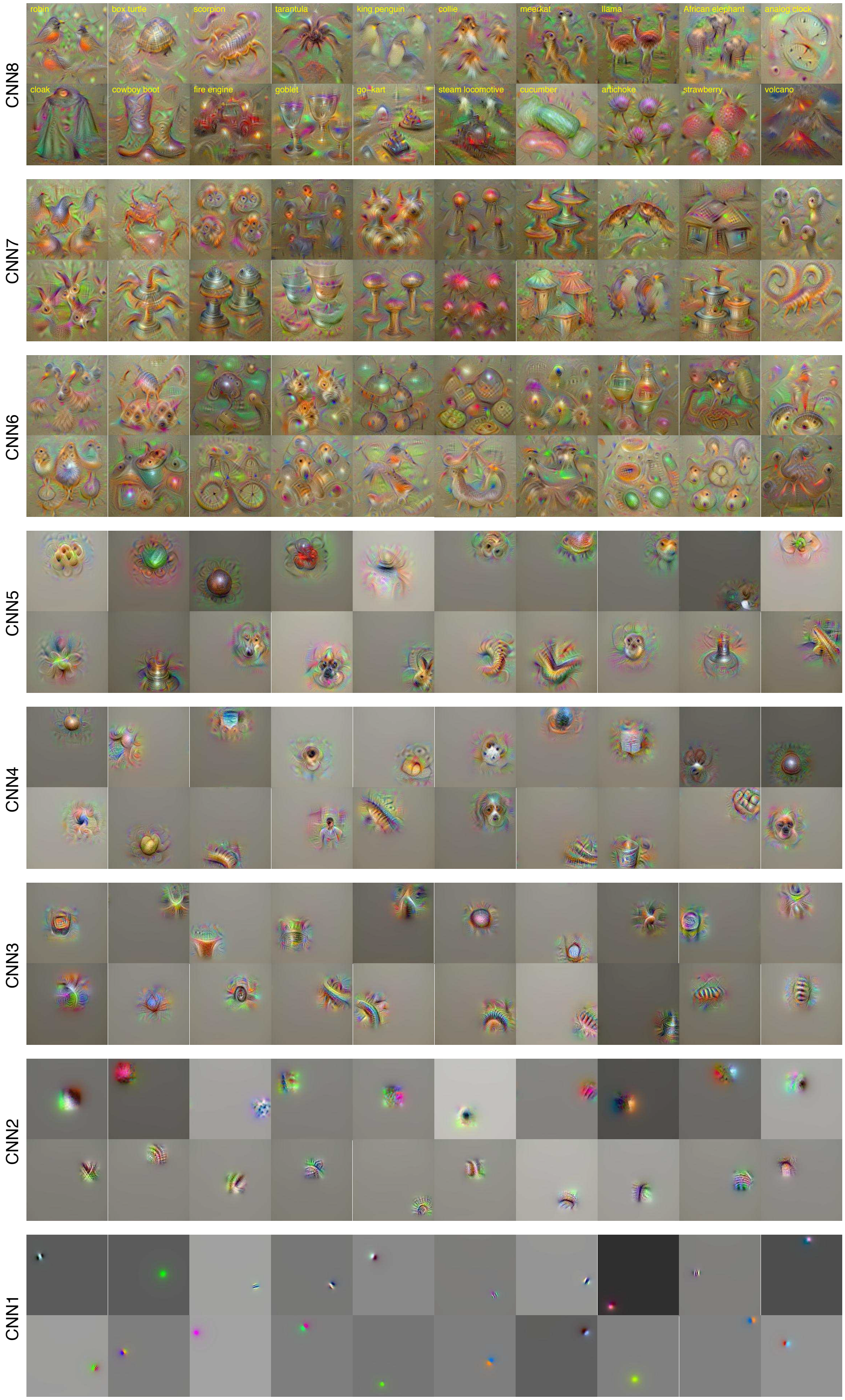
CNN8
robin
box turtle
scorpion
tarantula
king penguin
collie
meerkat
llama
African elephant
analog clock
cloak
cowboy boot
fire engine
goblet
go-kart
steam locomotive
cucumber
artichoke
strawberry
volcano
CNN7
CNN6
CNN5
CNN4
CNN3
CNN2
CNN1

Supplementary Figure 4 | Examples of preferred images for individual units in CNN layers. Examples of preferred images synthesized for each of the twenty randomly selected units in each CNN layer are shown. Category names of individual units in the CNN8 are shown at the top left of the images. Because the CNN6–8 are fully-connected layers, position information is lost for these layers.

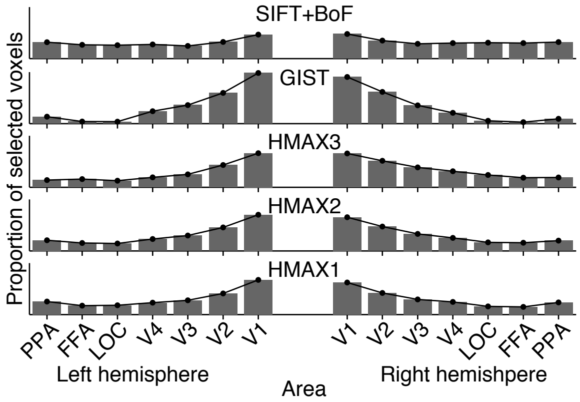

Supplementary Figure 5 | Distributions of selected voxels across individual subareas for HMAX, GIST, and SIFT+BoF. Distributions of selected voxels used for predictions of each visual feature type are shown for HMAX, GIST, and SIFT+BoF (averaged across five subjects, predicted from VC).

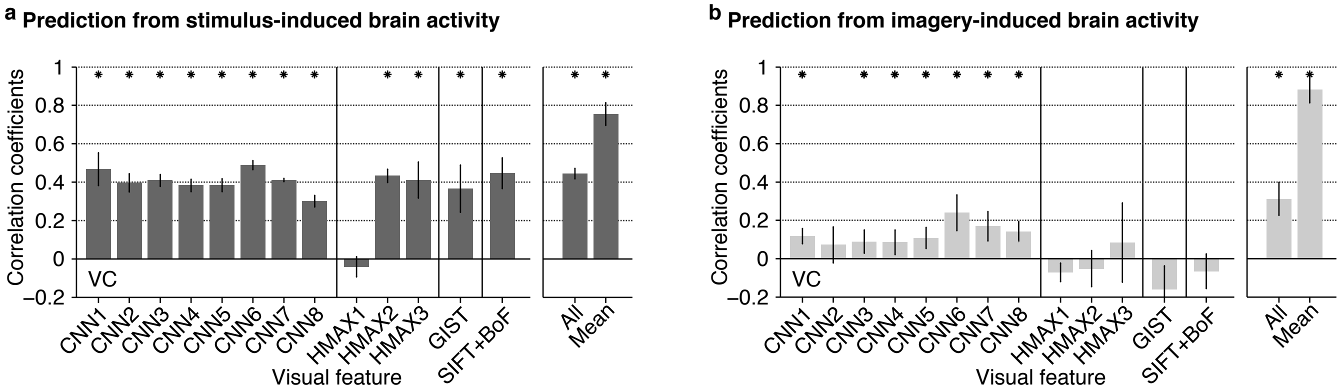

Supplementary Figure 6 | Relationship between category discriminability and prediction accuracy of category-average features. The same analysis described in Fig. 5c was performed with correlation coefficients between the values of the category-average features and the predicted features for seen and imagined conditions (predicted from VC by image feature decoders; cf., Fig. 6). Correlation coefficients between category discriminability and the category-average feature decoding accuracy are shown for the seen and imagined conditions (error bars, 95% CI across five subjects; asterisks, one-sided *t*-test after Fisher's *z*-transform, uncorrected $P < 0.05$). (**a**) Correlation coefficients obtained by predicting features from stimulus-induced brain activity. (**b**) Correlation coefficients obtained by predicting features from imagery-induced brain activity.

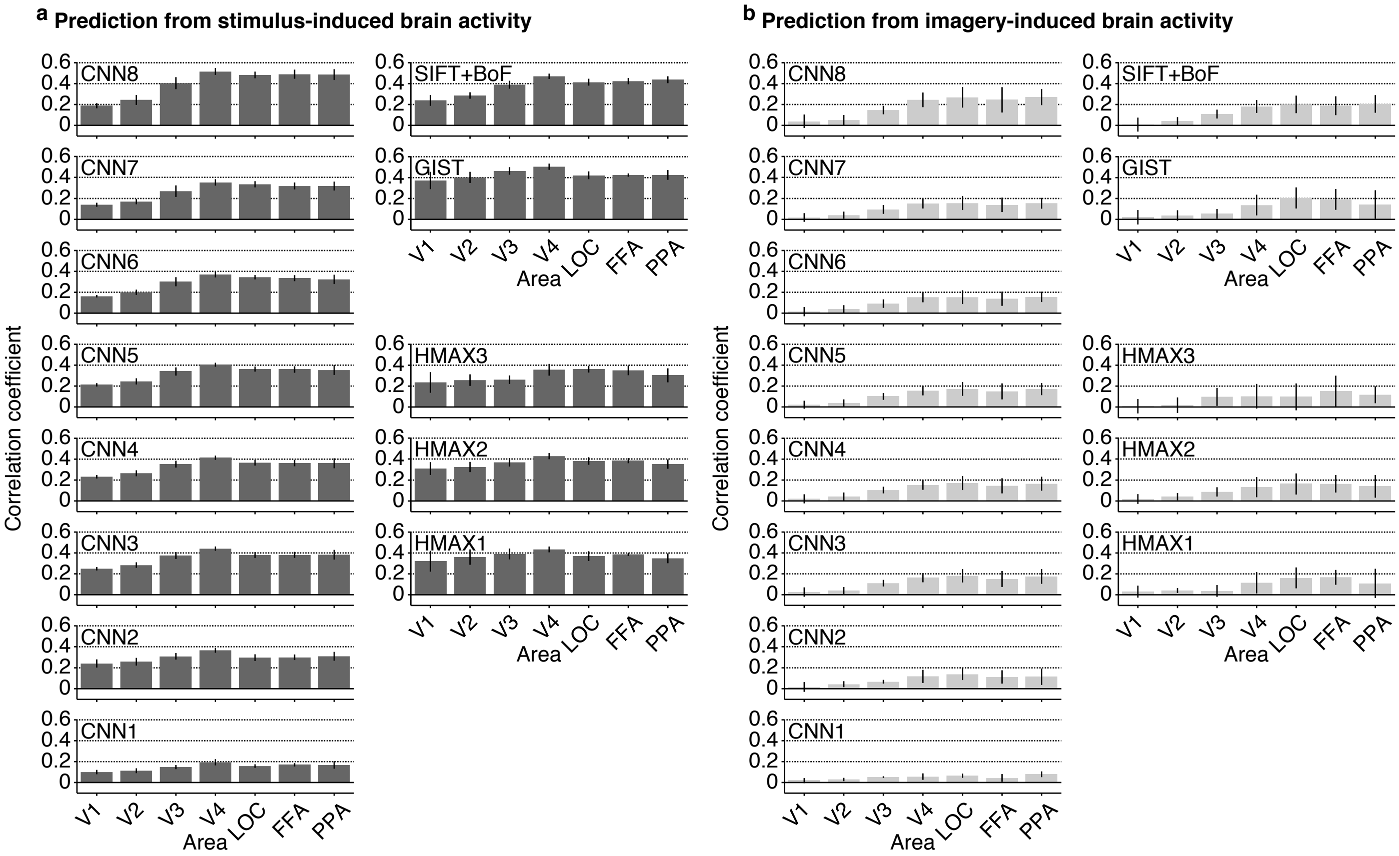

Supplementary Figure 7 | Prediction of category-average features from stimulus- and imagery-induced brain activity by category-average feature decoders. In the main analyses, while we used decoders that were trained to predict the feature values of the presented images (image feature decoders), it is possible to use the decoders trained to predict the category-average features (category-average feature decoders). Thus, here, we predicted category-average features using the category-average feature decoders (cf., Fig. 6 for the results produced by the image feature decoders). (**a**) Correlation coefficients with predicted features from stimulus-induced brain activity. (**b**) Correlation coefficients with predicted features from imagery-induced brain activity. Mean correlation coefficients are shown for each feature type/layer and ROI (error bars, 95% CI across five subjects). The results by the category-average feature decoders were qualitatively similar to those produced by the image feature decoders (Fig. 6), although slightly higher accuracy was obtained for the imagery-induced brain activity. This might be because the imagery-related brain activity was associated with multiple images imagined by the subjects.

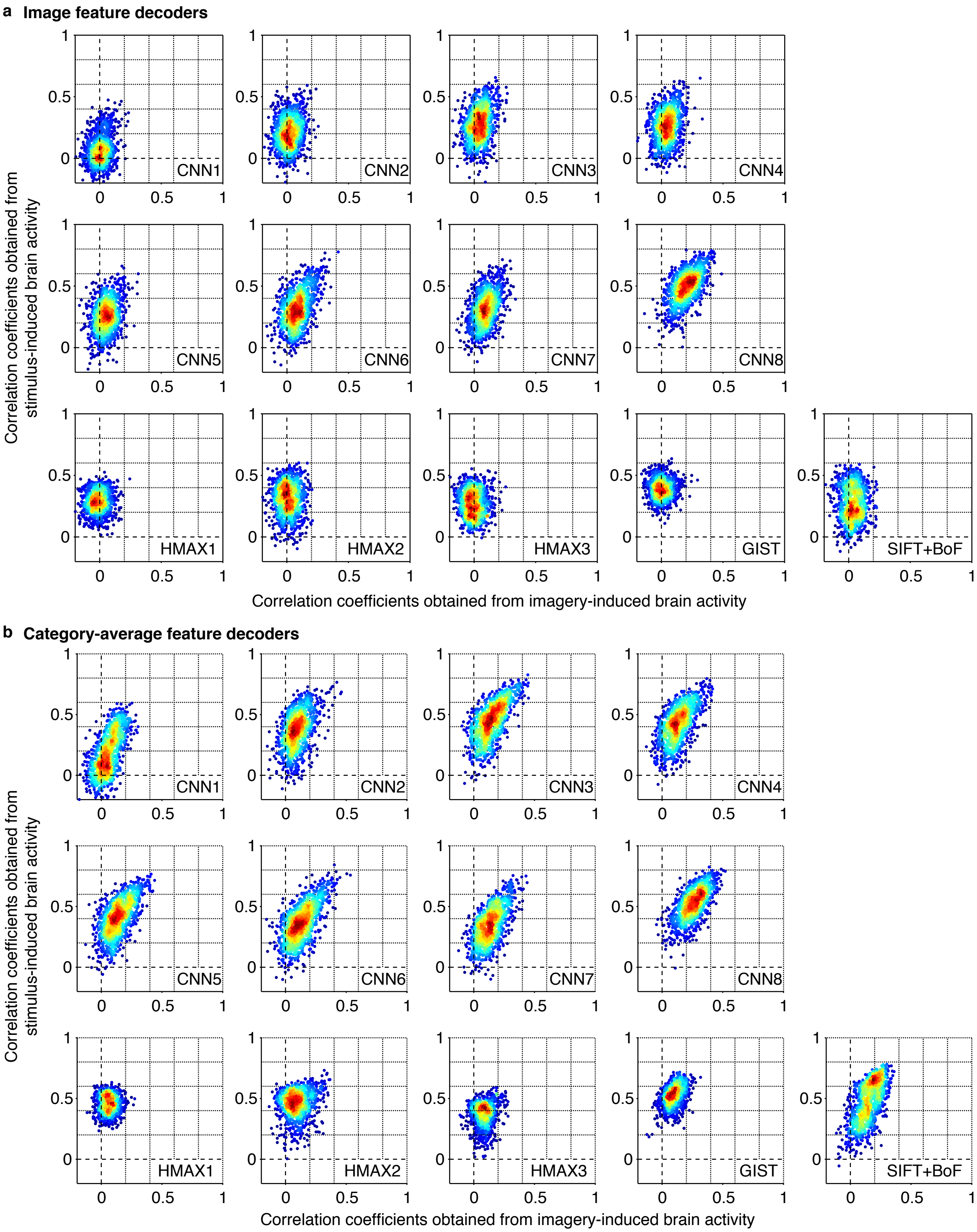
a Image feature decoders
b Category-average feature decoders
CNN1
CNN2
CNN3
CNN4
CNN5
CNN6
CNN7
CNN8
HMAX1
HMAX2
HMAX3
GIST
SIFT+BoF
Correlation coefficients obtained from stimulus-induced brain activity
Correlation coefficients obtained from imagery-induced brain activity
0
0.5
1

Supplementary Figure 8 | Distributions of correlation coefficients between predicted and category-average feature values for seen and imagined conditions. Scatterplots of correlation coefficients between the predicted and the category-average feature values for the seen (vertical axis) and imagined (horizontal axis) conditions are shown for ~1,000 feature units. (**a**) Distributions obtained by the image feature decoders. (**b**) Distributions obtained by the category-average feature decoders. Each dot denotes the averaged correlation coefficients across five subjects (predicted from VC) for each feature unit. The color indicates the density of the dots. Although the mean correlations ranged from approximately 0.1–0.5 for the seen condition (Fig. 6a and Supplementary Fig. 7a) and from approximately 0.0–0.2 for the imagined condition (Fig. 6b and Supplementary Fig. 7b), the correlations of individual units were broadly distributed. A subset of units with strong correlations may substantially contribute to object category decoding.

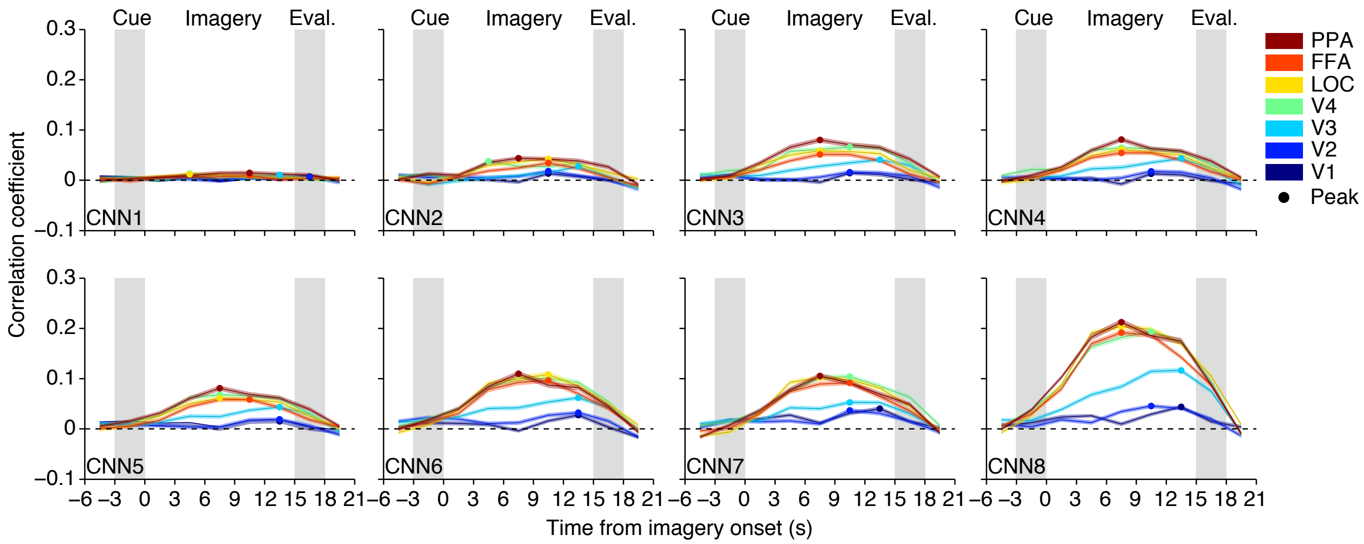

Supplementary Figure 9 | Time course of feature prediction from imagery-induced brain activity for individual CNN layers. At each time point/volume around the task period, correlation coefficients were calculated between the predicted and the category-average feature values for the series of test trials (averaged across five subjects; shaded areas, 95% CI across feature units; filled circles, peak timing). Predictions from imagery-induced brain activity in individual ROIs are shown for individual CNN layers.

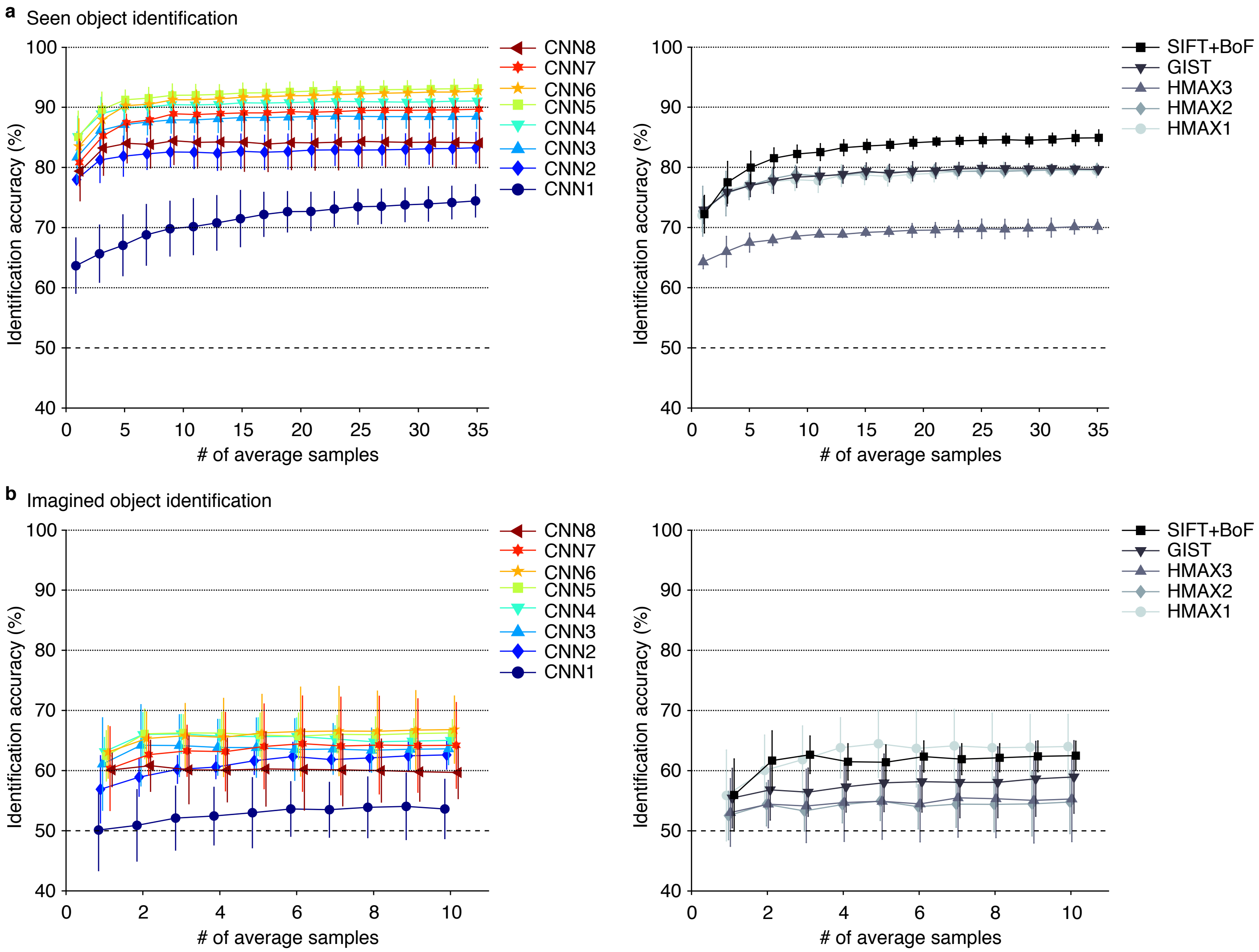

Supplementary Figure 10 | Identification accuracy as a function of the number of average samples. Identification accuracy is shown as a function of the number of average samples are shown (identification from two categories; predicted from VC by image feature decoders; error bars, 95% CI across five subjects; dashed line, chance level, 50%). (**a**) Seen object identification accuracy. The identification accuracy gradually improved with the number of average samples but saturated at fewer than ten samples for most feature types/layers. (**b**) Imagined object identification accuracy. Approximately equivalent accuracy was observed even without averaging multiple samples.

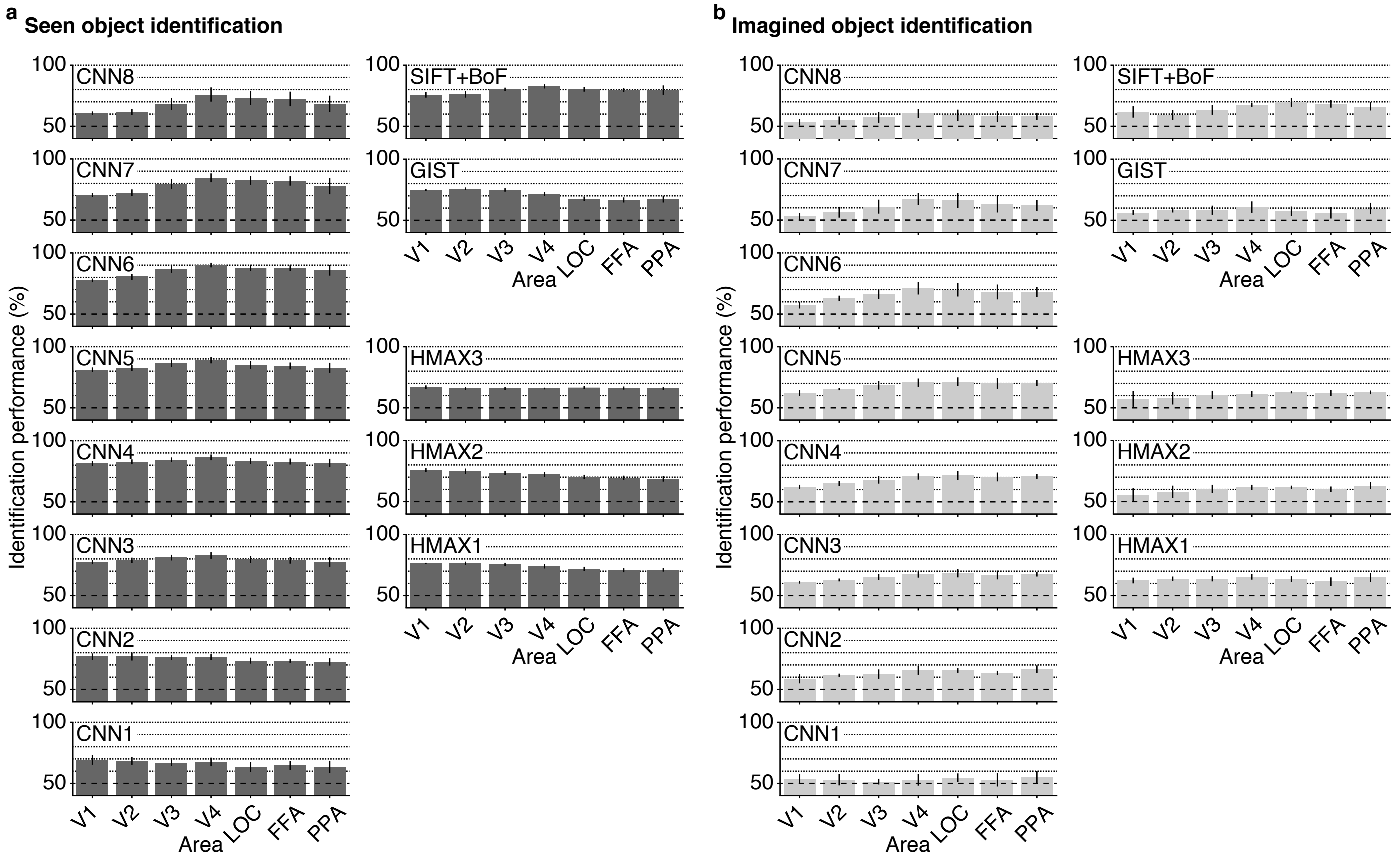

Supplementary Figure 11 | Identification accuracy for all combinations of feature types/layers and ROIs obtained by image feature decoders. Identification was performed for all combinations of one of the 50 test object categories and one of the 15,322 candidate categories (identification from two categories; error bars, 95% CI across five subjects; dashed line, chance level, 50%). (**a**) Seen object identification. (**b**) Imagined object identification. Both seen and imagined objects were successfully identified with most of the feature–ROI combinations (91 and 84 out of a total of 91 feature–ROI pairs for seen and imagined conditions, respectively; one-sided *t*-test, uncorrected $P < 0.05$). In seen object identification, the accuracy for higher-order features tended to be better with higher ROIs, while that for lower-order features tended to be better with lower ROIs, as observed in the image feature decoding accuracy (Fig. 3b). In contrast, in imagined object identification, all feature types/layers showed a similar trend, exhibiting flat or slightly elevated accuracy in higher ROIs.

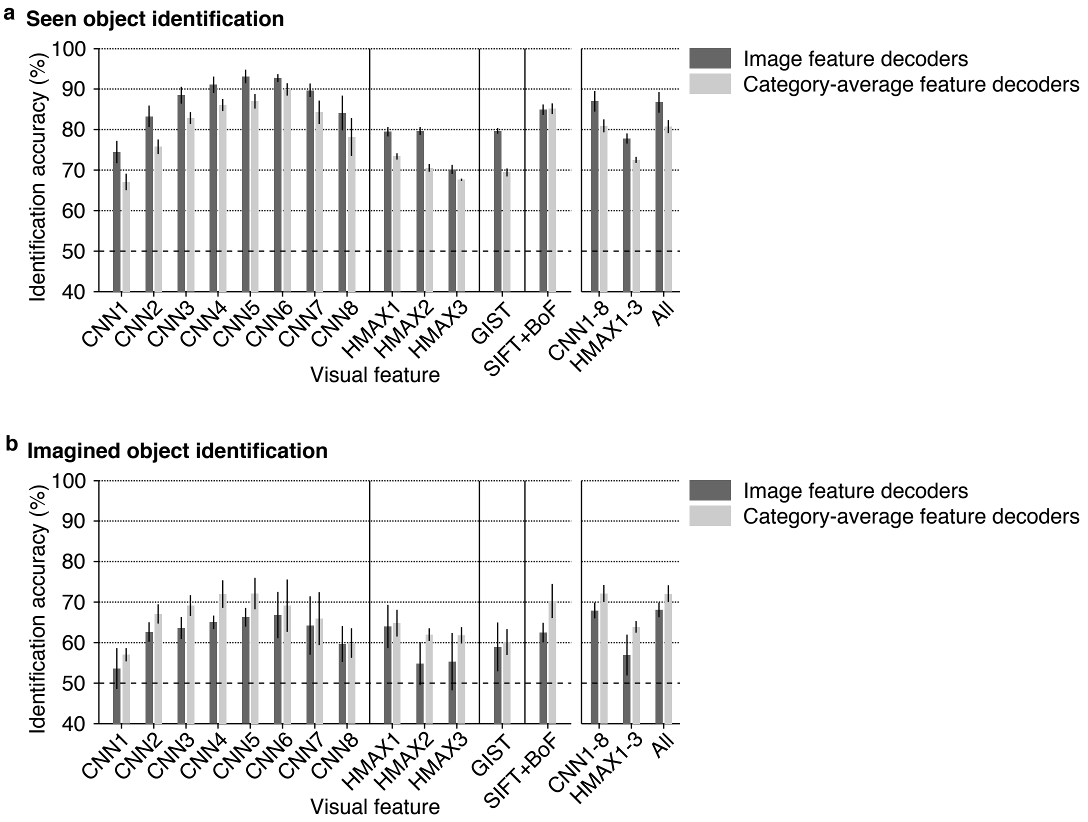

Supplementary Figure 12 | Identification accuracy by image feature decoders and category-average feature decoders. The same identification analyses shown in Fig. 10a and b (image feature decoders) were performed with the decoders trained to predict category-average features of presented images (category-average feature decoders; identification from two categories; error bars, 95% CI across five subjects; dashed line, chance level, 50%). (**a**) Seen object identification. (**b**) Imagined object identification. A similar pattern of accuracy across ROIs was observed from the two types of decoders. The overall accuracy for seen object identification tended to be higher with image feature decoders than with category-average feature decoders, while that for imagined object identification tended to be lower with image feature decoders than with category-average feature decoders.

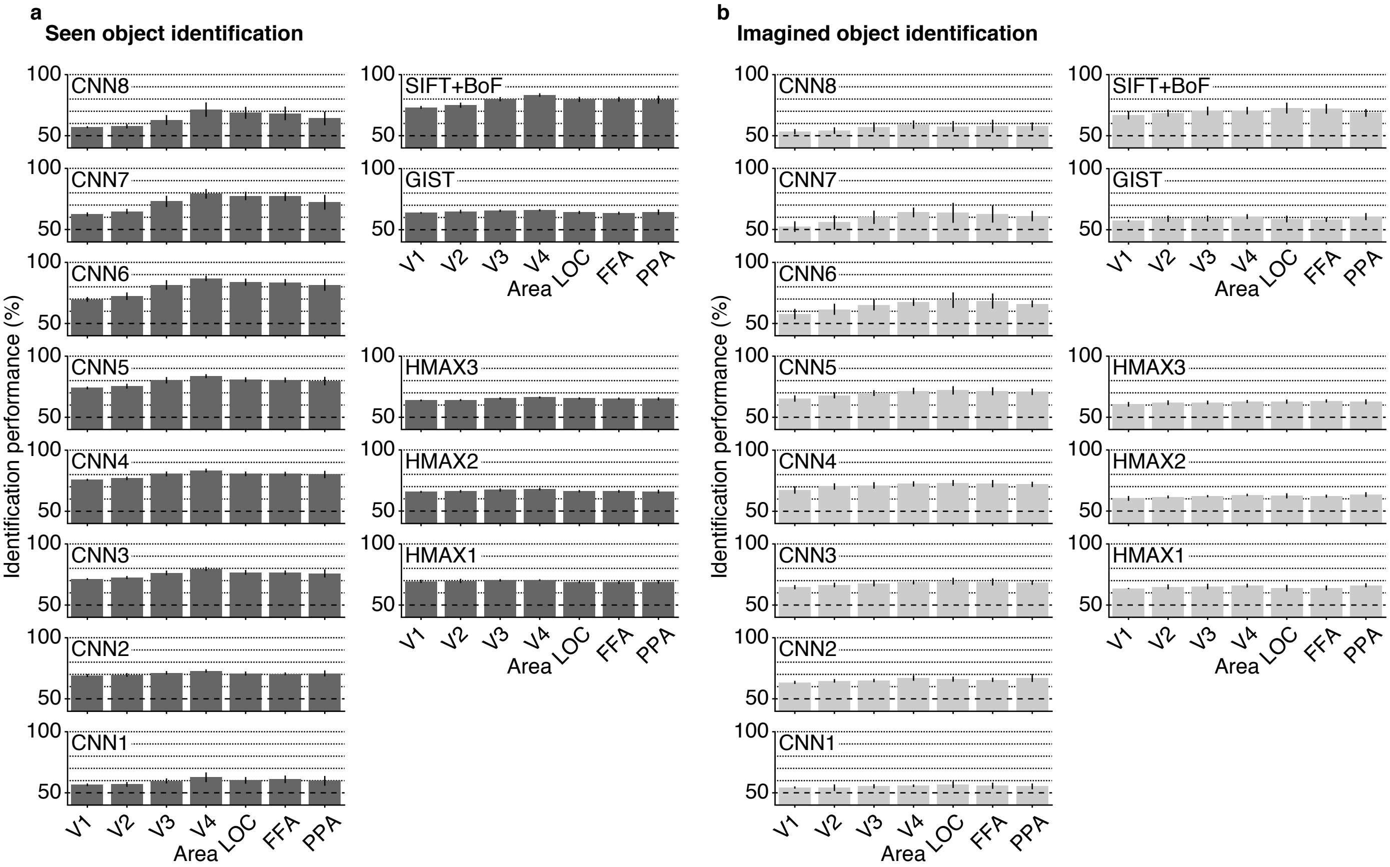

Supplementary Figure 13 | Identification accuracy for all combinations of feature types/layers and ROIs obtained by category-average feature decoders. The same identification analysis shown in Supplementary Fig. 11 was performed with the decoders trained to predict category-average features of the presented images (error bars, 95% CI across five subjects; dashed line, chance level, 50%). (**a**) Seen object identification accuracy. (**b**) Imagined object identification accuracy. Both seen and imagined objects were successfully identified at a statistically significant level with most of the feature–ROI combinations (91 and 90 out of a total of 91 feature–ROI pairs for seen and imagined conditions, respectively; one-sided *t*-test, uncorrected $P < 0.05$).

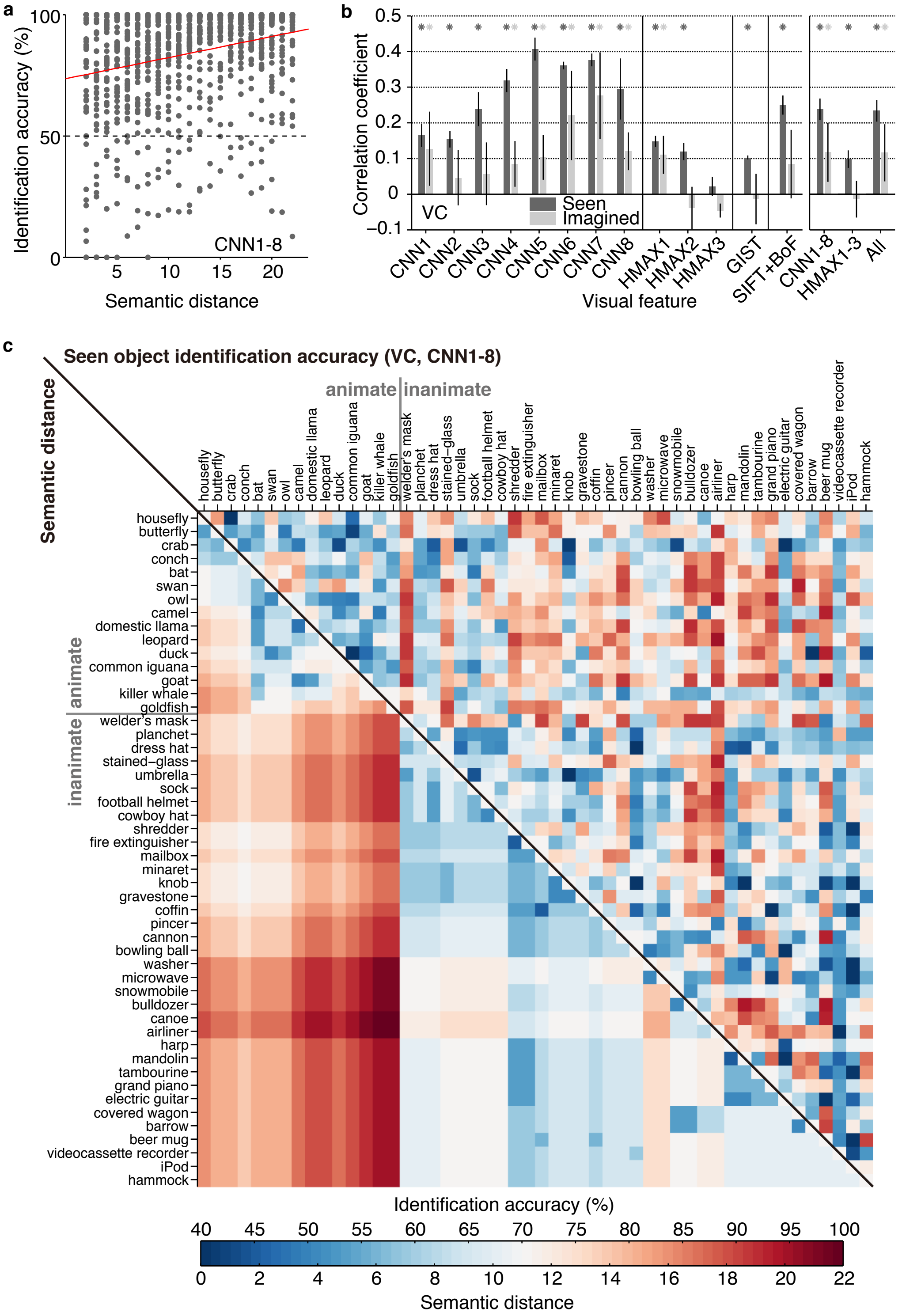
a
b
c
Identification accuracy (%)
Semantic distance
CNN1-8
Correlation coefficient
Visual feature
VC
Seen
Imagined
CNN1
CNN2
CNN3
CNN4
CNN5
CNN6
CNN7
CNN8
HMAX1
HMAX2
HMAX3
GIST
SIFT+BoF
CNN1-8
HMAX1-3
All
Seen object identification accuracy (VC, CNN1-8)
Semantic distance
animate
inanimate
housefly
butterfly
crab
conch
bat
swan
owl
camel
domestic llama
leopard
duck
common iguana
goat
killer whale
goldfish
welder's mask
planchet
dress hat
stained−glass
umbrella
sock
football helmet
cowboy hat
shredder
fire extinguisher
mailbox
minaret
knob
gravestone
coffin
pincer
cannon
bowling ball
washer
microwave
snowmobile
bulldozer
canoe
airliner
harp
mandolin
tambourine
grand piano
electric guitar
covered wagon
barrow
beer mug
videocassette recorder
iPod
hammock
Identification accuracy (%)
Semantic distance

Supplementary Figure 14 | Relationship between semantic distance and identification accuracy. Instead of evaluating mass identification accuracy by aggregating accuracies for all combinations of 50 test and 15,322 candidate categories (cf., Fig. 10a and b), identification accuracy was evaluated for each test category with candidate categories at a specified semantic distance from the test category (predicted from VC; averaged across five subjects) for (**a**) and (**b**). (**a**) Semantic distance versus seen identification accuracy from concatenated vectors of CNN1–8. Each dot in the scatterplot denotes the mean identification accuracy obtained by averaging identification accuracy for all combinations of one test category and candidate categories at a specified semantic distance to the test category. The solid red line indicates a fitted regression line. (**b**) Correlation coefficients between semantic distance and mean identification accuracy (asterisks, one-sided *t*-test after Fisher's *z*-transform, uncorrected $P < 0.05$). The identification accuracy and semantic distance tended to be positively correlated with each other, especially with high correlation coefficients for the mid-to-high level CNN layers (CNN4–8) under both the seen and imagined conditions. (**c**) A matrix of semantic distance and seen object identification accuracy among the 50 test categories. The semantic distance (lower triangle) and the seen object identification accuracy (upper triangle; CNN1–8; predicted from VC; averaged across five subjects) are shown for all pairs of the 50 test categories. Identification accuracies (upper triangle) were calculated with fMRI data from individual trials (without averaging multiple trials corresponding to the same category) so that the accuracy with each pair could be evaluated with many instances of identification. The matrix shows a moderate level of symmetry (with respect to the diagonal line), indicating a positive correlation between the semantic distance and the identification accuracy across the pairs. The segregation between animate vs. inanimate categories[1-4] can be observed in the identification accuracy as well as in the semantic distance.

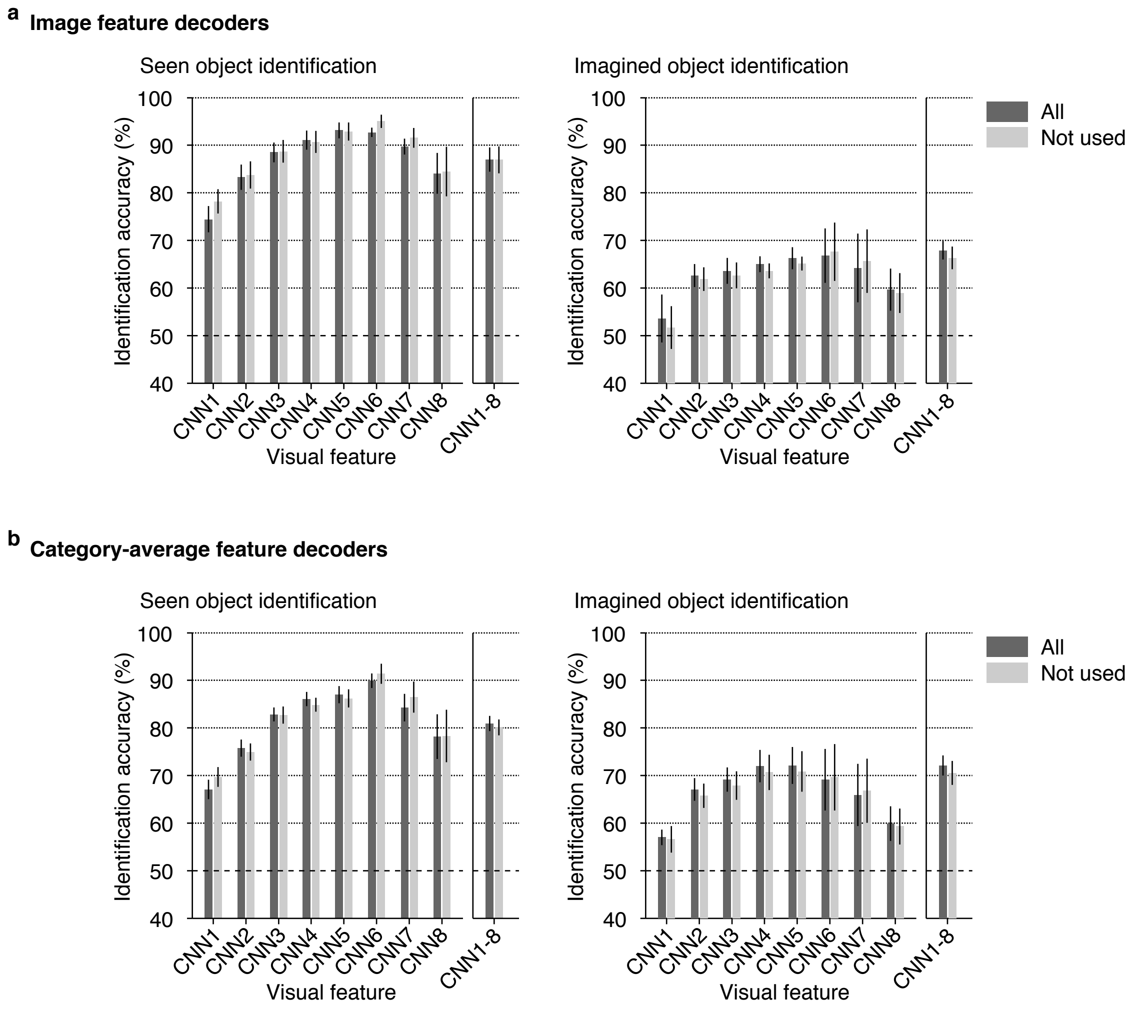

Supplementary Figure 15 | Identification accuracy for object categories not used for CNN model training. Mean identification accuracies for categories not used for CNN model training (*n* = 30) were evaluated and are shown with those for all 50 test categories (identification from two categories; predicted from VC; error bars, 95% CI across five subjects; dashed lines, chance level, 50%). (**a**) Identification accuracy obtained by image feature decoders. (**b**) Identification accuracy obtained by category-average feature decoders. Identification accuracies for categories not used for CNN model training were qualitatively consistent with those for all test categories under all conditions.

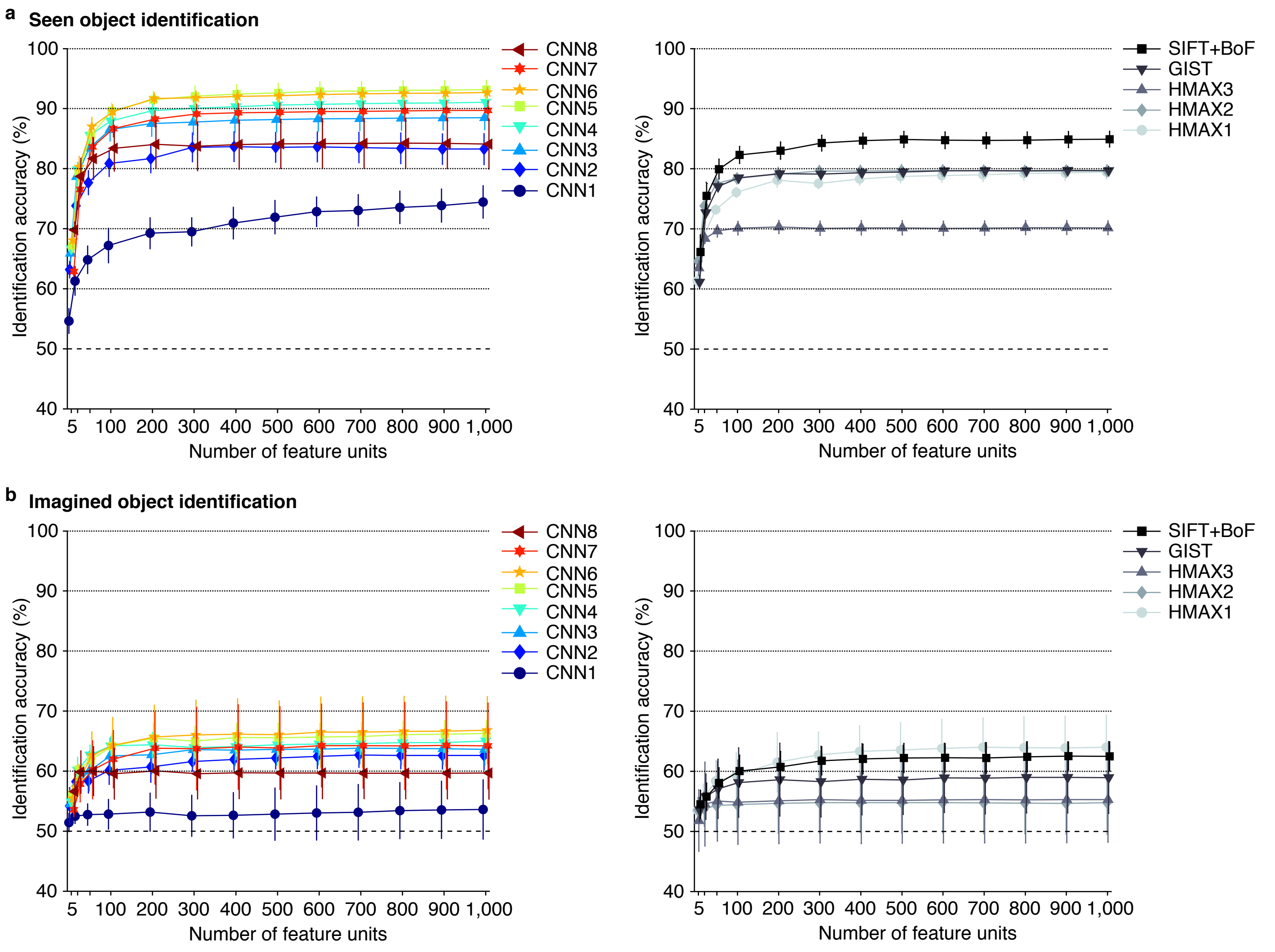

Supplementary Figure 16 | Identification accuracy as a function of the number of feature units. Identification was performed using a different number of feature units from each visual feature type/layer for all combinations of the 50 test object categories and 15,322 candidate categories (identification from two categories; predicted from VC by image feature decoders). The analysis was repeated 10 times for each number of feature units, and the accuracy was pooled across 10 repetitions of category candidate selection and 50 test samples (error bars, 95% CI across five subjects; dashed lines, chance level, 50%). (**a**) Seen object identification. (**b**) Imagined object identification. The accuracy for most visual features was saturated at a few hundred units. The accuracy trend across feature types/layers remained nearly constant across the number of feature units.

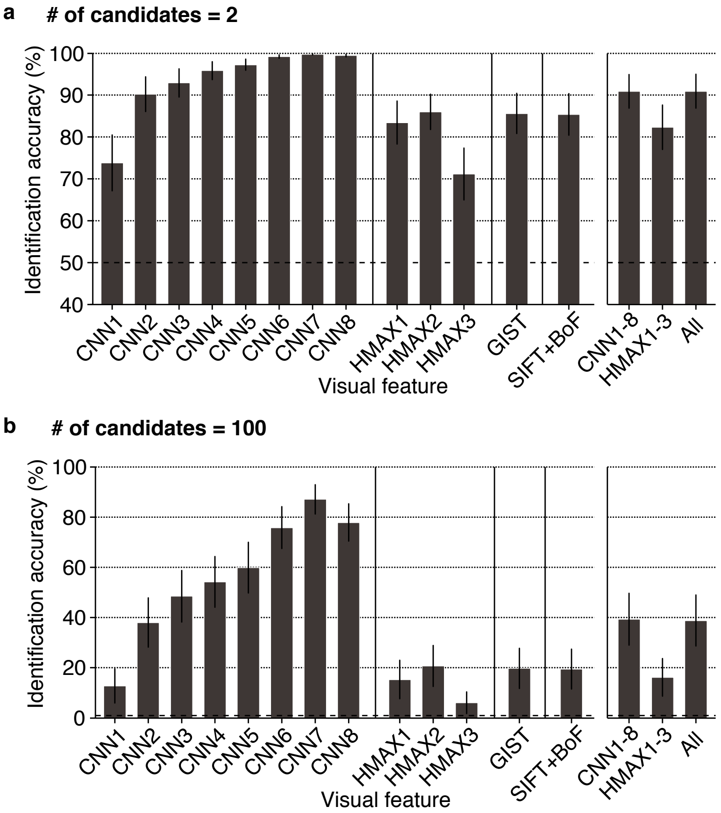

Supplementary Figure 17 | Identification accuracy with true image feature values (generic object recognition; GOR). The GOR identification accuracy for each visual feature type/layer is shown. The GOR accuracy is equivalent to the case in which image features are perfectly predicted from brain activity using image feature decoders. (**a**) Identification from two categories. Identification was performed for all combinations of one of the 50 test object categories and one of the 15,322 candidate categories (error bars, 95% CI across 50 test categories; dashed line, chance level, 50%). (**b**) Identification from 100 categories. Identification was repeated for 100 candidate sets of randomly selected 100 categories for each of the 50 test categories. The percentage of correct identifications was averaged across the candidate sets (error bars, 95% CI across 50 test categories; dashed line, chance level, 1%). The analysis showed a slightly poorer identification with CNN8 compared with CNN7. The high accuracy of original CNN features in the object recognition task may have contributed to the high accuracy of the CNN features in our generic decoding approach. Although the reason for the superior performance of CNN among other visual features continues to be debated in the field of computer vision, the acquisition of natural feature representations, which are shown as preferred images in Figure 4 and Supplementary Figure 4, may explain the high accuracy of CNN in object recognition.

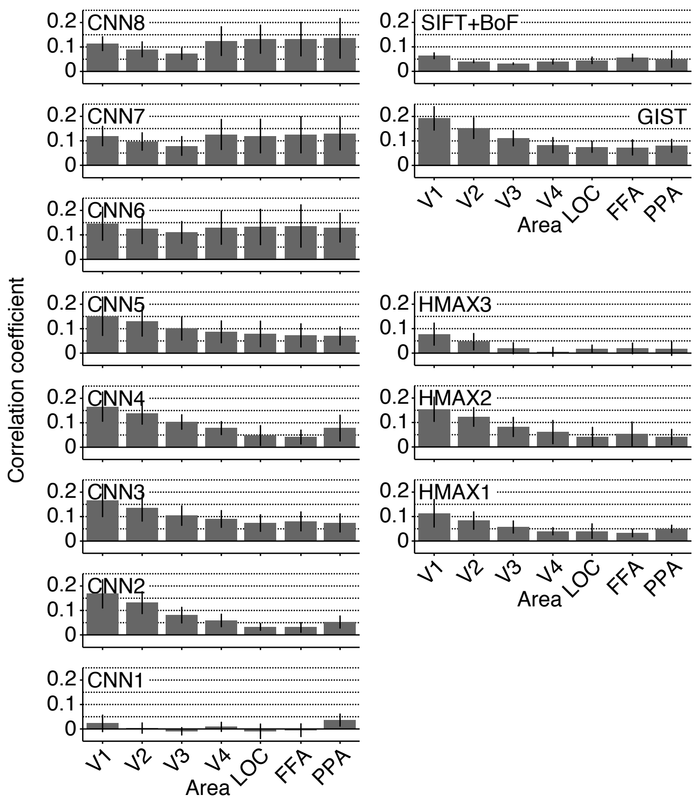

Supplementary Figure 18 | Voxel-wise encoding model prediction with image features. In the main analyses, we established relationships between brain activity and visual features via the visual feature predictions from brain activity patterns (i.e., decoding; cf., Fig. 3). However, it is also possible to use the voxel-wise encoding analysis[3-7], in which brain activity is predicted from visual features. Using the same dataset shown in Fig. 3, we first trained voxel-wise encoding models (sparse linear regression model[8]) to predict activity in the individual voxels from sets of visual feature values (~1,000 units for each feature type/layer) calculated from presented images. The voxel-wise encoding model accuracy of each voxel was then evaluated for each feature type/layer using Pearson's correlation coefficients between the observed and predicted voxel activity for the test images. The correlation coefficients were averaged over the voxels within each individual ROI. Mean correlation coefficients are shown for each combination of feature types/layers and ROIs (error bars, 95% CI across five subjects). High prediction accuracies were observed for lower/higher ROIs from lower/higher visual features, respectively. This trend was consistent with the results of the visual feature decoding approach (cf., Fig. 3), and with the findings of a previous study[7].

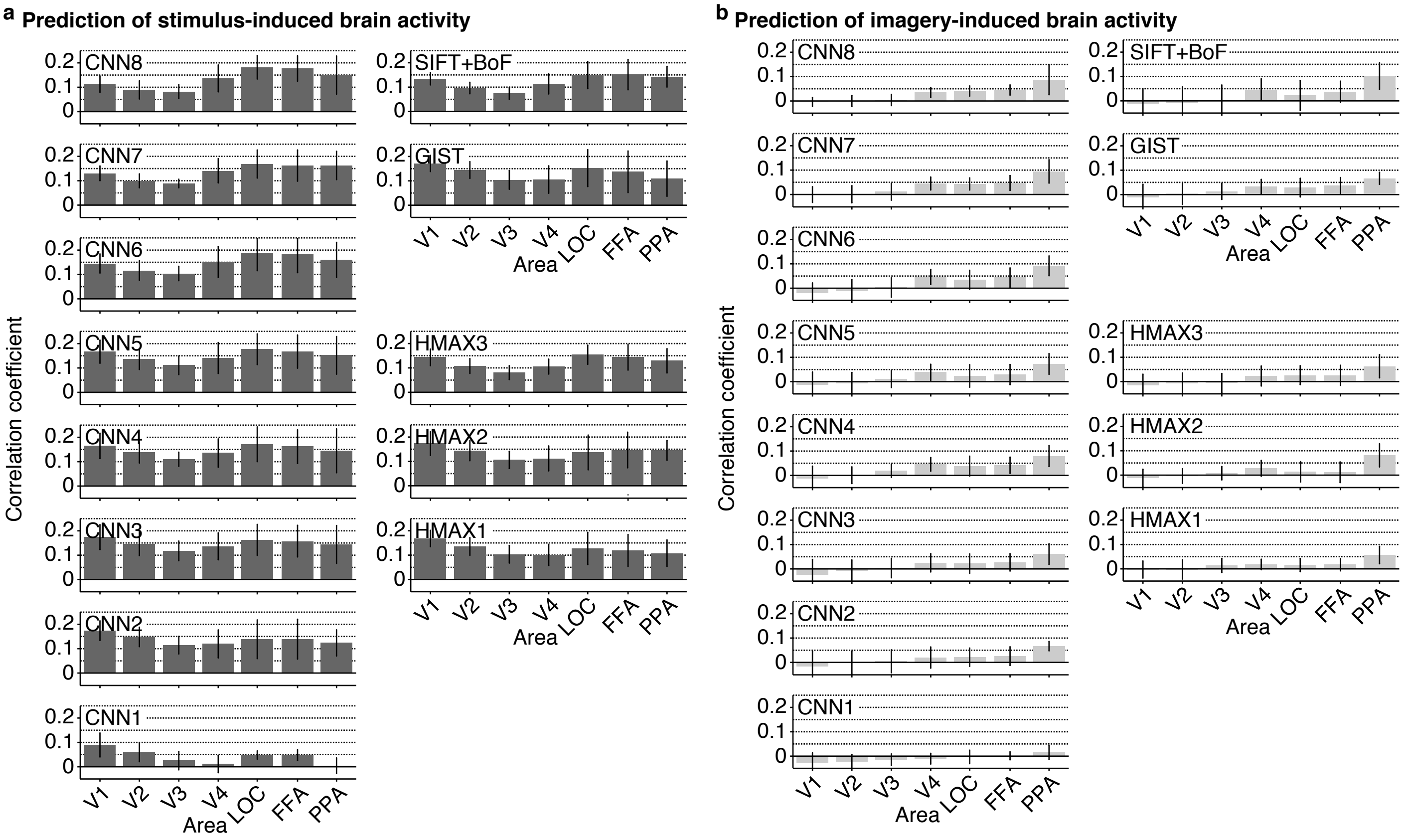

Supplementary Figure 19 | Voxel-wise encoding model prediction with category-average features. The same voxel-wise encoding analysis in Supplementary Fig. 18 was applied to the category-average features (cf., Fig. 6) to predict stimulus-induced and imagery-induced brain activity. (**a**) Correlation coefficients for stimulus-induced brain activity. (**b**) Correlation coefficients for imagery-induced brain activity. Mean correlation coefficients are shown for each feature type/layer and ROI (error bars, 95% CI across five subjects). Higher correlations were observed for combinations of mid-to-high level CNN features and mid-to-high level ROIs for both the perception and the imagery conditions, consistent with the results of the visual feature decoding approach (Fig. 6).

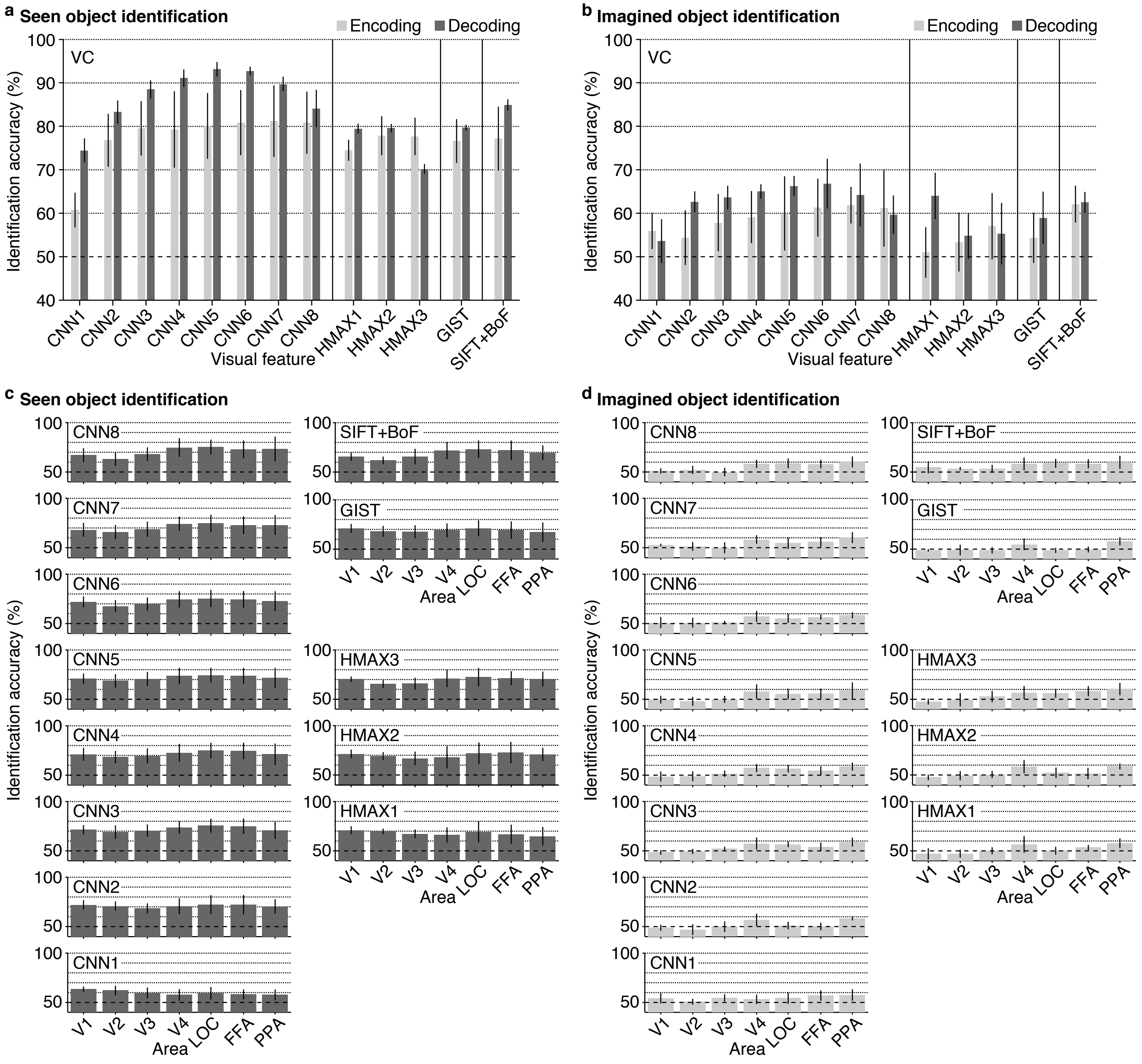

a Seen object identification
b Imagined object identification
Encoding
Decoding
VC
Identification accuracy (%)
Visual feature
CNN1
CNN2
CNN3
CNN4
CNN5
CNN6
CNN7
CNN8
HMAX1
HMAX2
HMAX3
GIST
SIFT+BoF
c Seen object identification
d Imagined object identification
Area
V1
V2
V3
V4
LOC
FFA
PPA

Supplementary Figure 20 | Identification accuracy via voxel-wise encoding models. We performed pairwise identification analyses using brain activity patterns predicted with voxel-wise encoding models (Supplementary Fig. 19). We created candidate brain activity patterns by converting the category-average feature vectors of 15,372 categories in ImageNet[9] to brain activity patterns using voxel-wise encoding models for each visual feature type/layer. The voxels showing the highest category discriminability (*F*-statistics, a ratio of inter- and intra-category variations of voxel activity in the training image session; 8 images from each of the 150 categories) were selected for the analyses of each ROI (100 voxels for individual areas; 200 voxels for VC). The pairwise identification analysis was then performed by calculating Pearson's correlation coefficients between the observed brain activity pattern (one of the 50 seen/imagined test categories) and two of the candidate brain activity patterns (one for the true and the other for a false category) and selecting the candidate category with a higher correlation coefficient. The analysis was performed using all combinations of the 50 test categories and candidate categories for each visual feature type/layer and ROI (error bars, 95% CI across five subjects; dashed line, chance level, 50%). (**a, b**) Seen/imagined object identification accuracies via the encoding and decoding approaches (predicted from VC). (**c, d**) Seen/imagined object identification accuracy via the encoding approach for all combinations of feature types/layers and ROIs. The identification analysis via the encoding approach showed high accuracy for mid-to-high visual features and mid-to-high ROIs, consistent with the results of the feature decoding approach. The results demonstrated the feasibility of generic object decoding via the encoding approach, while the overall accuracy was lower than that obtained by visual feature decoding. As shown in Supplementary Fig. 17, the visual feature space, especially in the mid- to high-level layers, was highly discriminable regarding object categories, while the brain space is generally much less discriminable (e.g., classification accuracy with fMRI responses to object categories[10,11]) due to the low signal to noise ratio and other factors. Thus, identification in visual feature space via the decoding approach may be more efficient than identification in brain space via the encoding approach.

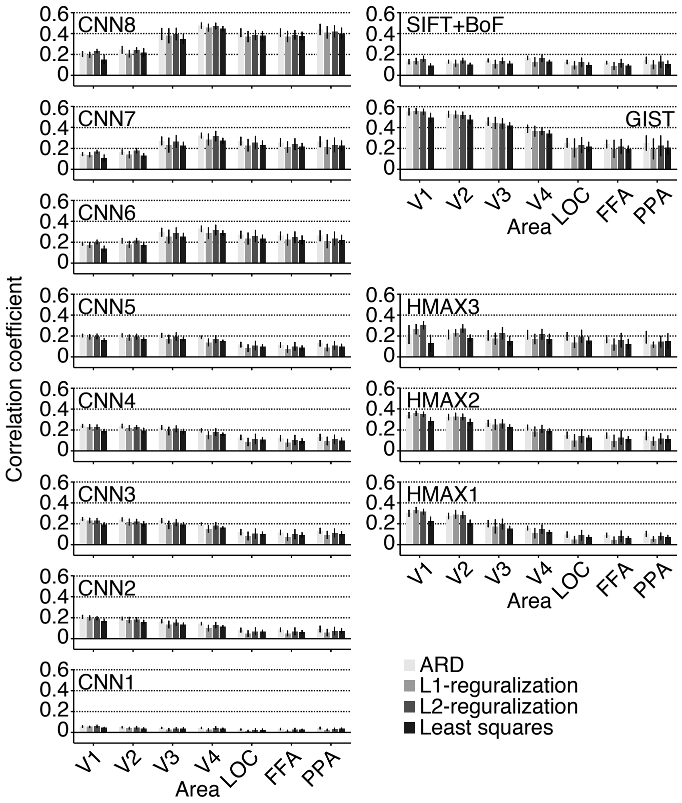

Supplementary Figure 21 | Image feature decoding by different linear regression models. While we used the Bayesian sparse linear regression models with the automatic relevance determination prior[8] (ARD model) in the main analyses, we also performed the feature decoding analyses using other types of linear regression models (see Methods: "*Visual feature decoding*"). Here, we compare the image feature decoding accuracies (cf., Fig. 3) obtained using the ARD model, the L1-/L2-reguralized linear regression models (Qian, J., Hastie, T., Friedman, J., Tibshirani, R. & Simon, N., Glmnet for Matlab, http://www.stanford.edu/~hastie/glmnet_matlab/, 2013), and the least squares linear regression model. Mean correlation coefficients are shown for each feature type/layer and ROI (error bars, 95% CI across five subjects). The decoding accuracies with these models are qualitatively similar, while the sparse models, especially the ARD model, showed relatively higher accuracy than the least squares model.

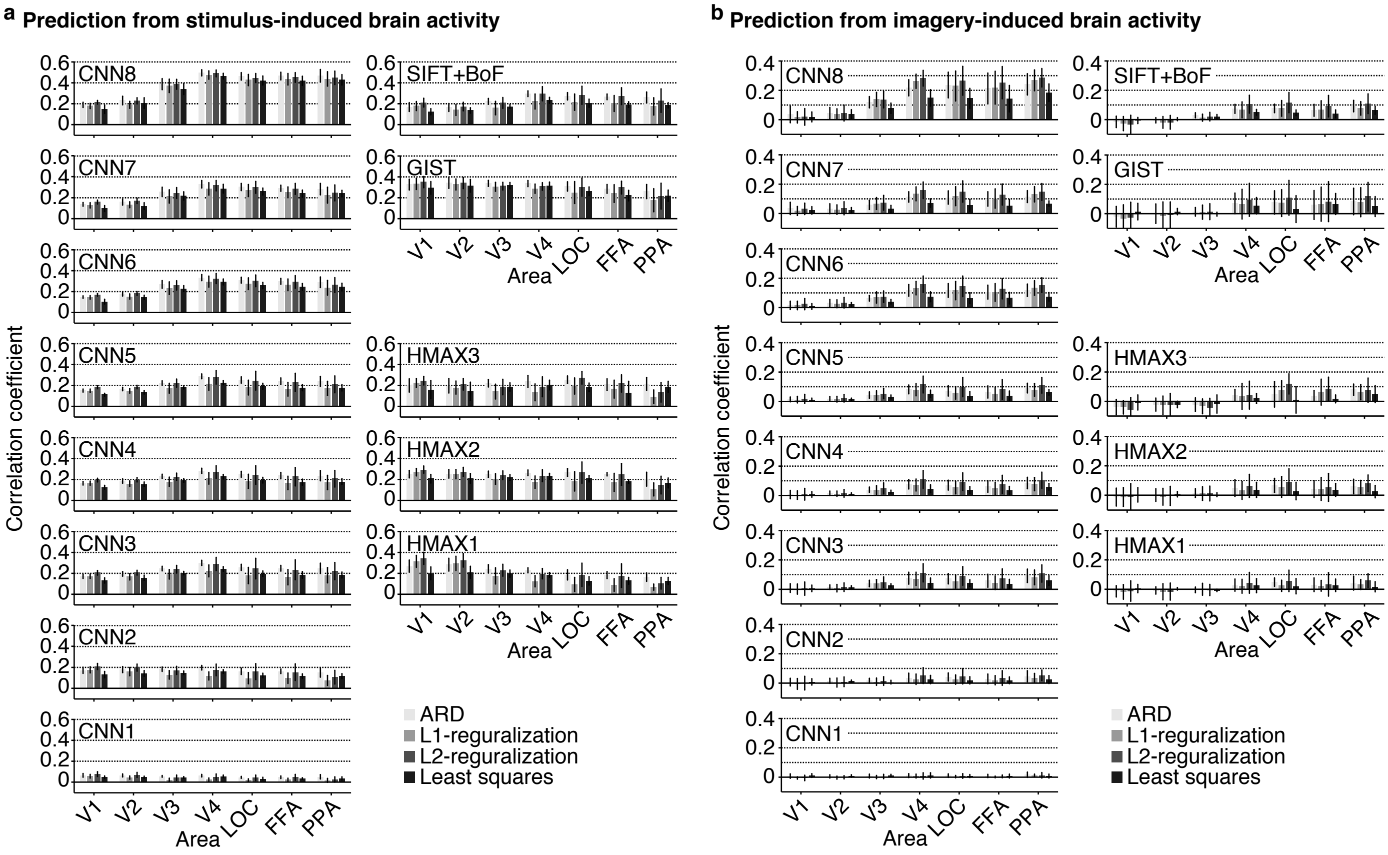

Supplementary Figure 22 | Prediction of category-average features by different linear regression models. Category-average features were predicted using different linear regression models as shown in Supplementary Fig. 21. (**a**) Correlation coefficients with predicted features from stimulus-induced brain activity. (**b**) Correlation coefficients with predicted features from imagery-induced brain activity. Mean correlation coefficients are shown for each feature type/layer and ROI (error bars, 95% CI across five subjects). This analysis also showed similar levels of accuracy across the models for both the perception and imagery conditions. Taken together with the results in Supplementary Fig. 21, these results indicate that image and category-average feature decoding can be reproduced with different linear regression models, supporting the robustness of our main results. The relative advantage of the sparse models suggests that regularization is useful to avoid overfitting.

Supplementary References

1. Downing, P. E., Chan, A. W. Y., Peelen, M. V., Dodds, C. M. & Kanwisher, N. Domain specificity in visual cortex. *Cereb. Cortex* **16,** 1453–1461 (2006).

2. Kriegeskorte, N. et al. Matching categorical object representations in inferior temporal cortex of man and monkey. *Neuron* **60,** 1126–1141 (2008).

3. Naselaris, T., Prenger, R. J., Kay, K. N., Oliver, M. & Gallant, J. L. Bayesian reconstruction of natural images from human brain activity. *Neuron* **63,** 902–915 (2009).

4. Huth, A. G., Nishimoto, S., Vu, A. T. & Gallant, J. L. A continuous semantic space describes the representation of thousands of object and action categories across the human brain. *Neuron* **76,** 1210–1224 (2012).

5. Kay, K. N., Naselaris, T., Prenger, R. J. & Gallant, J. L. Identifying natural images from human brain activity. *Nature* **452,** 352–355 (2008).

6. Naselaris, T., Olman, C. A., Stansbury, D. E., Ugurbil, K. & Gallant, J. L. A voxel-wise encoding model for early visual areas decodes mental images of remembered scenes. *Neuroimage* **105,** 215–228 (2015).

7. Güçlü, U. & van Gerven M. A. J Deep neural networks reveal a gradient in the complexity of neural representations across the ventral stream. *J. Neurosci*. **35,** 100005–100014 (2015).

8. Bishop, C. M. *Pattern Recognition and Machine Learning*. (Springer, New York, USA, 2006).

9. Deng, J. et al. Imagenet: A large-scale hierarchical image database. *IEEE CVPR* (2009).

10. Haxby, J. V. et al. Distributed and overlapping representations of faces and object in ventral temporal cortex. *Science* **293,** 2425–2430 (2001).

11. Reddy, L., Tsuchiya, N. & Serre, T. Reading the mind's eye: Decoding category information during mental imagery. *Neuroimage* **50,** 818–825 (2010).
